# Trends in mortality from gastrointestinal, hepatic, and pancreatic cancers in the United States: A comprehensive analysis (1999–2020)

**DOI:** 10.1002/jgh3.13064

**Published:** 2024-04-15

**Authors:** Hassam Ali, Rizwan Ishtiaq, Brandon Tedder, Joshua Zweigle, Romina Nomigolzar, Dushyant S Dahiya, Vishali Moond, Amir Humza Sohail, Pratik Patel, Debargha Basuli, Hans L Tillmann

**Affiliations:** ^1^ Department of Gastroenterology, Hepatology & Nutrition ECU Health Medical Center, Brody School of Medicine Greenville North Carolina USA; ^2^ Department of Internal Medicine University of Connecticut Health Center Farmington Connecticut USA; ^3^ Department of Internal Medicine ECU Health Medical Center, Brody School of Medicine Greenville North Carolina USA; ^4^ Ross University School of Medicine Bridgetown Barbados; ^5^ Department of Internal Medicine Central Michigan College of Medicine Saginaw Michigan USA; ^6^ Department of Internal Medicine Saint Peter's University Hospital, Robert Wood Johnson Medical School New Brunswick New Jersey USA; ^7^ Department of Surgery NYU Langone Health Long Island New York USA; ^8^ Department of Gastroenterology Mather Hospital, Hofstra University Zucker School of Medicine Port Jefferson New York USA

**Keywords:** continental population groups, gastrointestinal neoplasms, health status differences, mortality, time factors

## Abstract

**Background and Aim:**

This study investigates temporal trends in gastrointestinal cancer‐related mortality in the United States between 1999 and 2020, focusing on differences by sex, age, and race.

**Methods:**

We investigated the Centers for Disease Control and Prevention Wide‐Ranging Online Data for Epidemiologic Research multiple causes of death database (Years 1999–2020) for gastrointestinal cancer‐related mortality with a focus on the underlying cause of death.

**Results:**

A total of 3 115 243 gastrointestinal cancer‐related deaths occurred from 1999 to 2020. The overall age‐adjusted mortality rate decreased from 46.7 per 100 000 in 1999 to 38.4 per 100 000 in 2020. The average annual percent change (AAPC) for the study period was −0.9% (95% CI: −1.0%, −0.9%, *P* < 0.001), with no significant difference in AAPC between the sexes but some difference between races and related to individual cancers. African Americans and Asian Americans, and Pacific Islanders experienced a greater decrease in mortality compared with Whites. Mortality rates for American Indian and Alaskan Native populations also decreased significantly from 1999 to 2020 (*P* < 0.001). There were significant declines in esophageal, stomach, colon, rectal, and gallbladder cancer‐related mortality but increases in the small bowel, anal, pancreatic, and hepatic cancer‐related mortality (*P* < 0.001), with variation across different sexes and racial groups.

**Conclusion:**

While overall gastrointestinal cancer‐related mortality declined significantly in the United States from 1999 to 2020, mortality from some cancers increased. Furthermore, differences between sexes and racial groups underscore crucial differences in gastrointestinal cancer mortality, highlighting areas for future research.

## Graphical abstract

From 1999 to 2020, gastrointestinal cancer‐related mortality in the United States generally declined, with the age‐adjusted mortality rate decreasing from 46.7 to 38.4 per 100,000. Despite this overall reduction, disparities persist with varying rates of decline among different racial groups and increases in mortality for cancers of the small bowel, anus, pancreas, and liver. Significant declines were noted in esophageal, stomach, colon, rectal, and gallbladder cancers, highlighting variations by sex and race.

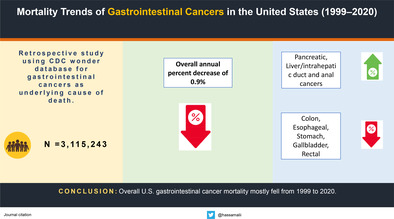

## Introduction

Gastrointestinal (GI) cancers constitute a significant public health issue worldwide, contributing a substantial fraction of all annual cancer‐related fatalities, representing 26% of worldwide cancers and 35% of cancer‐related deaths.^1^ In 2018, in the United States alone, there were 284 844 new cases diagnosed and 155 090 deaths attributed to GI malignancies.^2^ The most prevalent GI cancers include colorectal, liver, esophageal, pancreatic, and stomach cancers. Colorectal cancer persistently ranks as the third most common cancer worldwide.^3^

Discerning high‐risk subpopulations is paramount as individualizing treatment protocols based on a patient's specific characteristics, encompassing race and genetic profile, may be essential. Advancements in surveillance, diagnosis, and treatment have improved outcomes for some patients, but mortality rate differences by sex and race have endured.^4^

Evidence indicates that demographic factors such as sex influence GI cancer incidence and mortality rates. For instance, males were reported to be twice as susceptible to GI cancers as females, with some variation among the different GI cancers.^1^ Additionally, racial and ethnic differences in GI cancer incidence and mortality are also present.^5^ Two studies reported African Americans exhibit higher rates of colorectal and liver cancer than other racial groups, whereas Asians have higher rates of stomach cancer.^6^, ^7^ Such differences may be attributable to variations in genetic susceptibility, lifestyle factors like diet, or socioeconomic factors such as healthcare access.

Identifying changing trends and differences in GI cancer mortality can provide invaluable insights into devising efficacious interventions and screening methodologies to alleviate the burden of these malignancies. Previous studies have been limited to a narrow period, relatively small sample sizes, and less focus on differences or specific cancers.^8^, ^9^ This study summarizes trends in all GI cancer‐related mortality and differences within the United States, specifically examining shifts in mortality rates and inequalities among diverse demographic subgroups.

## Methods

### Data source

We retrieved de‐identified data from the Centers for Disease Control and Prevention Wide‐Ranging Online Data for Epidemiologic Research (CDC WONDER) multiple causes of death database (Years 1999–2020) for gastrointestinal cancer‐related mortality with a focus on the underlying cause of death.^10^ Cancer patients were identified using International Classification of Diseases 10th Revision Clinical Modification codes, as given in Appendix S1, Supporting information. Anonymized data and materials can be found in the CDC WONDER database^10^ The deaths attributed to gastrointestinal cancers as a primary cause of death on nationwide death certificates were selected using the Underlying Cause‐of‐Death Public Use records. This approach has been validated and employed in studies examining mortality trends previously.^11^, ^12^ As the CDC WONDER database comprises publicly accessible, anonymized data, the study was not subject to local Institutional Review Board approval.

### Data extraction

Data were extracted from the latest available data, including the years 1999–2020, and included cancer‐specific deaths, population sizes, sex (male, female), and race defined as African Americans (AA), Whites, American Indian or Alaska Native (AI/AN), and Asian Americans and Pacific Islanders (AAPI). The category “Hispanic origin” was not analyzed to avoid bias, as Hispanic ethnicity is likely to be misclassified on death certificates, as reported in prior literature.^13^

### Statistical analysis

The CDC WONDER database reports crude mortality rates (CMRs) and age‐adjusted mortality rates (AAMRs) per 100 000 population. CDC Wonder database calculates CMRs by dividing the number of cancer‐related deaths by the corresponding US population for a specific year. AAMRs, on the other hand, are calculated by standardizing cancer‐related deaths to the 2000 US population. The Joinpoint software (v4.9.1.0; National Cancer Institute) assessed temporal trends in the Annual percent change (APC), which represented the change in mortality occurring during a specific period, expressed as a percentage, and averaged over the specified period. Log‐linear regression models were fitted to evaluate temporal trends in CMRs and AAMRs.

Joinpoint employed a Monte Carlo permutation test to identify time points, referred to as “joinpoints,” where the trend or magnitude changed significantly from the preceding one, using a t‐test, as used previously.^14^, ^15^ The simplest model was fitted by applying the smallest trend segments or periods. A Poisson distribution was assumed and a maximum of three joinpoints were pre‐specified. This method is commonly used to minimize bias when assessing trends in incidence or mortality rates, as used previously.^14^, ^16^

The final models were utilized to estimate the APC and Average Annual Percent Change (AAPC) along with their corresponding 95% confidence intervals (CIs) and *P*‐values. To assess differences in trends between sex, age groups, and races over time, we conducted a nonparallel pairwise comparison using Joinpoint regression analysis, and results were reported as AAPC difference with 95% CI and *P*‐values. Statistical significance was established at *P* ≤ 0.05. Following best practices and guidelines, we did not present or publish death counts of nine or fewer or death rates based on counts of nine or fewer in any form of visual or tabular representation, such as figures, graphs, maps, or tables.^10^

## Results

**Figure 1:**
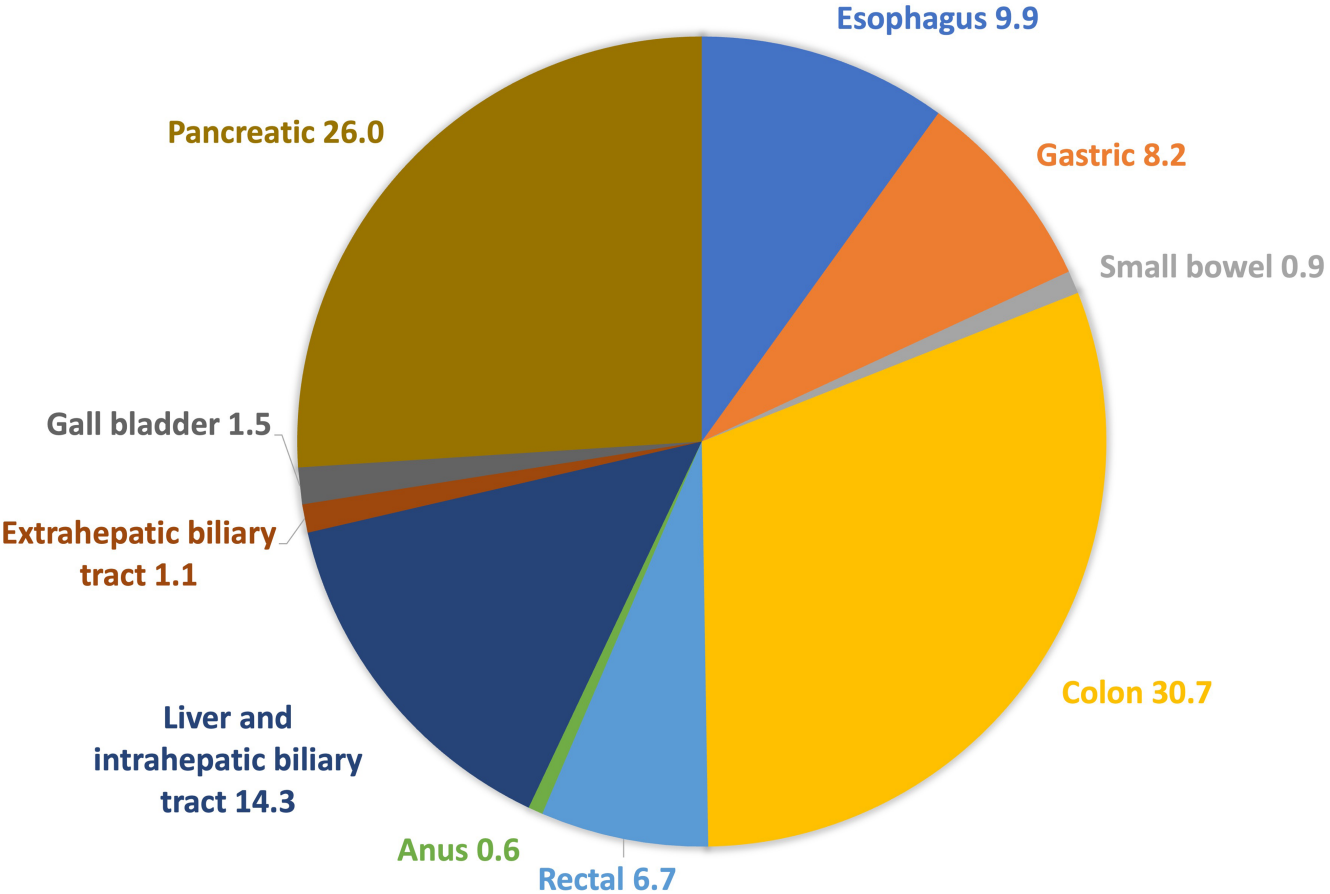
Distribution of gastrointestinal cancers related mortality from 1999 to 2020 in the United States. All numbers are in percentages (%).

**Figure 2:**
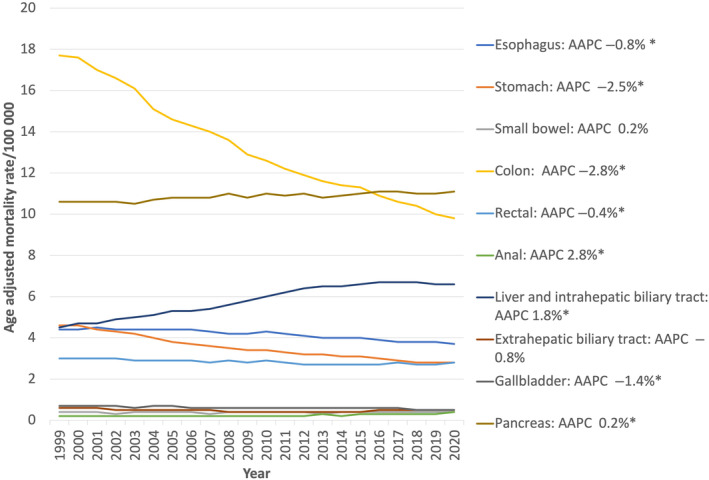
Joinpoint regression analysis of total gastrointestinal cancers related mortality from 1999 to 2020 in the United States, stratified by sex. AAPC, average annual percent change; *P < 0.05.

**Figure 3:**
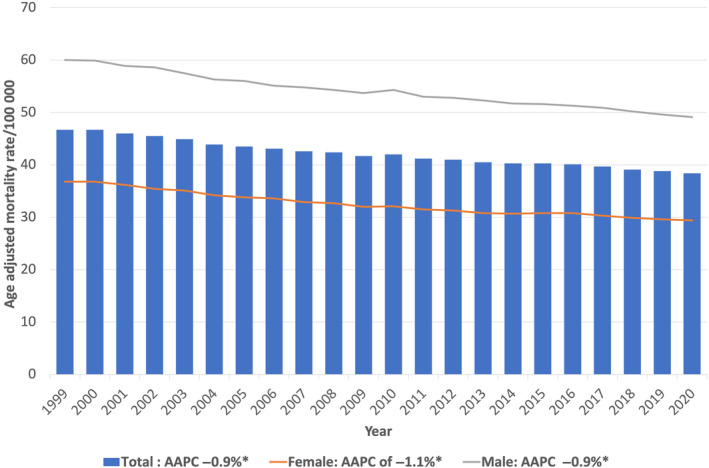
Joinpoint regression analysis of total gastrointestinal cancers related mortality from 1999 to 2020 in the United States, stratified by race. AAPC, average annual percent change; *P < 0.05.

Between 1999 and 2020, there were 3 115 243 deaths related to gastrointestinal cancers (5.48% of all deaths and 24% of all cancer‐related deaths reported during the same period). The highest number of gastrointestinal cancer‐related deaths was due to colon cancer (30.7%), followed by pancreatic cancer (26.0%) and liver and intrahepatic biliary tract cancers (14.3%) (Figs. 1 and 2). The overall AAMR decreased from 46.7 per 100 000 in 1999 to 38.4 per 100 000 in 2020, with an AAPC of −0.9% (95% CI: −1.0%, 0.9%, *P* < 0.001). There was a significantly larger decrease in GI cancer mortality rates from 1999 to 2006 (APC of −1.3%, 95% CI: −1.5%, −1.1%, *P* < 0.001) than in more recent years from 2006 to 2020 (APC of −0.8%, 95% CI: −0.8%, −0.7%, *P* < 0.001) (Table 1, Fig. 3).

**Table 1:** Gastrointestinal cancer‐related deaths in the United States from 1999 to 2020

Location	Total deaths (*n*)	AAMR (1999)^†^	AAMR (2020)^†^	AAMR (1999–2020)^†^	AAPC (1999–2020) with 95% CI	*P*‐value
Total	3 115 243	46.7	38.4	41.8	0.9% (−1.0%, −0.9%)	<0.001
Colon	957 232	17.7	9.8	13	−2.8% (−3.0%, −2.7%)	<0.001
Esophagus	30 919	4.4	3.7	4.1	−0.8% (−1.0%, −0.9%)	<0.001
Stomach	254 105	4.6	2.8	3.4	−2.5% (−2.7%, −2.3%)	< 0.001
Rectal	208 926	3.0	2.8	2.8	−0.4% (−0.7%, −0.1%)	0.003
Pancreas	810 628	10.6	11.1	10.9	0.2% (0.2%, 0.3%)	<0.001
Gallbladder	45 470	0.7	—	0.6	−1.4% (−1.8%, −1.0%)	<0.001
Liver and intrahepatic biliary system	446 959	4.5	6.6	5.9	1.8% (1.6%, 2.1%)	<0.001
Anus	18 300	0.2	0.4	0.2	2.8% (1.4%, 4.3%)	<0.001
Small bowel	28 380	0.4	0.4	0.4	0.2% (−0.5%, 0.9%)	0.547
Extrahepatic biliary system	35 324	0.6	0.5	0.5	−0.8% (−1.6%, 0.1%)	0.091

AAMR, age‐adjusted mortality rate; AAPC, average annual percent change; APC, annual percent change; CI, confidence interval.

^†^ Per 100 000; overall rates were age‐adjusted to the 2000 US standard population.

### Sex‐based differences in overall GI cancer mortality

For females, the AAMR decreased from 36.8 per 100 000 in 1999 to 29.4 per 100 000 in 2020, with an AAPC of −1.1% (*P* < 0.001), while for males, the AAMR decreased from 60 per 100 000 in 1999 to 49.1 per 100 000 in 2020, with an AAPC of −0.9% (*P* < 0.001). Time segment analysis for different periods showed a significant decrease in mortality rates from 1999 to 2009, both for females (APC of −1.4%, 95% CI: −1.6%, −1.3%, *P* < 0.001) and males (APC of −1.3%, 95% CI: −1.5%, −1.0%, *P* < 0.001) and from 2009 to 2020 females (APC of −0.8%, 95% CI: −0.9%, −0.6%, *P* < 0.001) and males (APC of −0.8%, 95% CI: −0.9%, −0.7%, *P* < 0.001) (Fig. 3).

There was a significant decline for both females and males in cancer‐related mortality for the following cancers: esophageal, stomach, colon, rectal, gallbladder, and extrahepatic biliary system from 1999 to 2020 (Appendix S2). As with overall GI cancer mortality rates, there was an uptrend in the pancreatic, anal, and liver/intrahepatic biliary system cancer‐related mortality for the study period for both females and males (APC between 3.4% and 0.2%). The direction of cancer trends along with AAPCs for the study period is described in Table 2. Individual trends for each cancer with individual join points are further described in detail in Appendix S1.

**Table 2:** Average annual percent change (AAPC) (95% confidence interval) of mortality trends in gastrointestinal cancers from 1990 to 2020

	Females	Males	White	African Americans	Asian Americans and Pacific Islanders	American Indian or Alaska Natives
Total	−1.1% (−1.2%, −1.0%)	−0.9% (−1.0%, −0.9%)	−0.8% (−0.9%, −0.7%)	−1.7% (−1.7%, −1.6%)	−1.6% (−1.9%, −1.4%)	−1.0% (−1.3%, −0.8%)
Colon	−2.9%^†^ (−3.4%, −2.4%)	−2.9%^†^ (−3.3%, −2.5%)	−2.8%^†^ (−3.0%, −2.6%)	−3.2%^†^ (−3.3%, −3.0%)	−1.8%^†^ (−2.7%, −0.9%)	−1.9%^†^ (−2.4%, −1.3%)
Esophagus	−1.4%^†^ (−1.5%, −1.2%)	−0.8%^†^ (−1.0%, −0.6%)	−0.2%^†^ (−0.5%, 0.0%)	−4.5%^†^ (−4.7%, −4.3%)	−1.3%^†^ (−1.7%, −0.9%)	−1.6%^†^ (−2.5%, −0.6%)
Stomach	−2.3%^†^ (−2.6%, −2.0%)	−2.9%^†^ (−3.0%, −2.7%)	−2.5%^†^ (−2.7% to −2.2%)	−3.3%^†^ (−3.4% to −3.1%)	−3.7%^†^ (−4.0% to −3.5%)	−3.2%^†^ (−3.9% to −2.5%)
Rectal	−0.6%^†^ (−0.8%, −0.5%)	−0.5%^†^ (−0.3%, −0.7%	−0.5%^†^ (−0.6%, −0.3%)	−0.6% (−1.2%, 0.0%)	−0.5%^†^ (−0.9%, −0.2%)	−0.9% (−2.1%, 0.3%)
Pancreas	0.2%^†^ (0.1%, 0.3%)	0.2% (−0.0%, 0.4%)	0.3%^†^ (0.3%, 0.4%)	−0.3%^†^ (−0.4%, −0.2%)	−0.0% (−0.3%, 0.3%)	−0.1% (−0.7%, 0.6%)
Gallbladder	−1.5%^†^ (−1.9%, −1.1%)	−1.2%^†^ (−1.8%, −0.7%)	−2.0%^†^ (−3.0%, −0.9%)	0.1% (−0.6%, 0.8%)	−1.2%^†^ (−2.2%, −0.1%)	N/A
Liver and intrahepatic biliary tract	1.6%^†^ (1.2%, 2.0%)	1.8%^†^ (1.6%, 2.0%)	2.1%^†^ (1.9%, 2.3%)	1.2%^†^ (0.6%, 1.7%)	−1.5%^†^ (−2.0%, −0.9%)	1.3%^†^ (0.6%, 1.9%)
Anal	3.4%^†^ (2.6%, 4.2%)	2.5%^†^ (1.0%, 4.1%)	3.2%^†^ (1.9%, 4.5%)	2.7% (−0.3%, 5.8%)	N/A	N/A
Small bowel	0.8% (−0.1%, 1.8%)	0.4% (−1.0% to 1.9%)	0.2% (−1.6%, 2.1%	1.1%^†^ (0.3% to 1.8%)	N/A	N/A
Extrahepatic biliary	−1.6%^†^ (−2.6%, −0.7%)	−1.2%^†^ (−2.4%, −0.0%)	−0.5% (−1.6%, 0.7%)	0.4% (−0.6%, 1.3%)	−1.0%^†^ (−2.0%, −0.0%)	N/A

N/A, not available due to less number of cases.

^†^ Significant trend from 1999 to 2020.

### Overall GI cancer mortality rates by race

**Figure 4:**
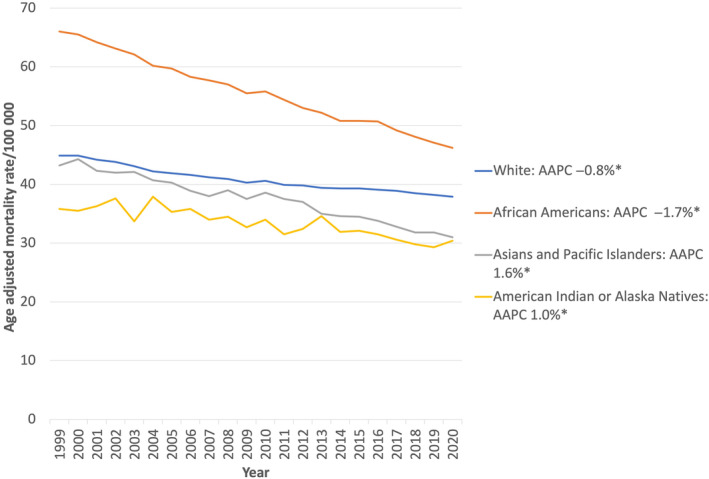
Joinpoint regression analysis of individual gastrointestinal cancers related mortality from 1999 to 2020 in the United States, stratified by cancer location. AAPC, average annual percent change; *P < 0.05.

The overall difference associated with GI cancer mortality was also seen when exploring mortality by race (Fig. 4). The decrease was highest for AA, with an AAMR of 66 per 100 000 in 1999 to 46.2 per 100 000 in 2020, with an AAPC of −1.7% (*P* < 0.001), followed by AAPI who experienced the AAMR decreased from 43.2 per 100 000 in 1999 to 31 per 100 000 in 2020 with an AAPC of −1.6% (*P* < 0.001). For AI/AN, the AAMR decreased from 35.8 per 100 000 in 1999 to 30.4 per 100 000 in 2020, with an AAPC of −1.0% (*P* < 0.001). For the White populations, the AAMR decreased from 44.9 per 100 000 in 1999 to 41.9 per 100 000 in 2020, with an AAPC of −0.8% (*P* < 0.001).

### Individual GI cancers and their mortality rates by race

Mortality rates significantly decreased for colon, esophagus, and stomach cancers across all racial groups (Appendix S2, Table 2). Rectal cancer declined among Whites, AA, and AAPI but remained stable in AI/AN. Gallbladder cancer trends decreased for Whites and AAPI but remained unchanged for AA (due to small numbers difference could not be assessed for AI/AN). Pancreatic cancer increased among Whites, declined in AA, and stayed constant for AAPI and AI/AN. Liver and intrahepatic biliary tract cancer rates rose among Whites, AA, and AI/AN, but dropped for AAPI. Anal cancer increased for Whites and remained stable for AA (due to small numbers, difference could not be assessed for AAPI and AI/AN). Small bowel cancer was stable for Whites and increased for AA (due to small numbers, difference could not be assessed for AAPI and AI/AN). Extrahepatic biliary tract cancer remained constant for Whites and AA but decreased in AAPI (due to small numbers, difference could not be assessed for AI/AN) (Appendix S3, Table 2). Individual trends for each cancer with individual join points are further described in detail in Appendix S1.

### Age‐based differences for GI cancers

For total cancers of the GI tract, the 25–44 age group experienced a decrease in mortality with a CMR of 4.5 per 100 000 in 1999 to 4.3 per 100 000 in 2020 and an AAPC of −0.3% (95% CI: −0.5 to −0.1, *P* = 0.002). In contrast, the 45–64 age group experienced an increase in mortality with a CMR of 50.4 per 100 000 in 1999 to 53.2 per 100 000 in 2020 and an AAPC of 0.3% (95% CI: 0.1 to 0.4, *P* < 0.001). For the 65+ age group, there was a significant decrease in mortality, with a CMR of 268.5 per 100 000 in 1999 to 203.3 per 100 000 in 2020 and an AAPC of −1.3% (95% CI: −1.5 to −1.2, *P* < 0.001).

Liver and intrahepatic biliary tract cancer mortality rates showed opposing trends for different age groups, with a significant decrease for the 25–44 age group but a significant increase for the 45–64 age group and the 65+ age group.

Similarly, for pancreatic cancer, the 25–44 age group had a significant decrease, while the 45–64 age group saw a significant increase. The 65+ age group experienced no significant change in mortality rates from pancreatic cancer. Additional trends are described in Appendix S1.

## Discussion

Between 1999 and 2020, the United States experienced over 3 million GI cancer‐related deaths, with colon cancer accounting for 30.7% of these fatalities. Although the AAMR significantly decreased during this period, with an overall AAPC of −0.9%, some differences were observed when examining the data based on sex, age, and race. Mortality rates for small bowel, pancreatic, anal, and hepatic/intrahepatic biliary system cancers increased, while rates for esophageal, stomach, colon, rectal, gallbladder, and extrahepatic biliary system cancers decreased. Racial differences were also observed, with AA, AAPI, and AI/AN population experiencing more significant decreases in mortality rates than White populations; however, there still was higher cancer mortality for the non‐White population overall for the study period.

Like our analysis, a study comparing mortality trends for stomach cancer among adolescents and young adults in four countries, including the USA, reported declining death rates from 1990 to 2019.^17^

Several factors may contribute to the observed trends in gastrointestinal cancer‐related mortality. The decline in certain cancer‐related mortalities can be attributed to advancements in early detection, prevention strategies, and treatment options.^18^, ^19^ For example, the widespread adoption of colonoscopy screening has significantly decreased colon cancer mortality.^20^, ^21^ Of note, while there has been an increase in colon cancer being diagnosed in younger people,^22^ resulting in a change to recommend colon cancer screening at an earlier age,^23^ that increase in colon cancer incidence among younger adults has not translated into higher colon cancer mortality rates. Evidence suggests that sigmoidoscopies reduce CRC incidence and mortality more in men than women due to women having more frequent proximal lesions that cannot be seen with a sigmoidoscope.^24^, ^25^ This difference may be partly attributable to women's willingness to undergo more colonoscopies than sigmoidoscopies.^26^

Fairly widespread helicobacter eradication treatment, when detected, results in the elimination of one of the most significant risk factors for stomach cancer^27^ Improved treatments and early detection have decreased stomach cancer‐related mortality.^28^, ^29^ Conversely, increases in mortality rates for some gastrointestinal cancers, such as pancreatic and hepatic cancers, are concerning.^30^, ^31^ This trend for pancreatic cancer may be due to the increased prevalence of risk factors such as diabetes and obesity^32^, ^33^ and a lack of progress in pancreatic cancer‐specific primary prevention, early detection, and treatment.^34^ The rise in hepatic cancer‐related mortality is concerning as this comes on the backbone of HBV having been controllable for two decades and HCV basically being curable in all patients. Direct‐acting antivirals (DAAs) have enabled cure rates of over 95%.^35^ After antiviral against HBV reduced related mortality, the introduction of DAAs significantly decreased HCV‐related liver cirrhosis, liver failure, and hepatocellular carcinoma (HCC), the United States' most common primary liver cancer.^35^ Due to their high efficacy and ability to halt the progression of liver disease, DAAs are anticipated to reduce the rates of HCC caused by HCV in the upcoming years. However, with the widening epidemic of obesity, NASH‐associated HCCs are expected to continue to increase. The magnitude of the obesity epidemic likely outpaces the reduction in HCV‐associated HCC. Also, an increase in alcohol use may further increase HCC development.

Various factors, including differences in healthcare access, socioeconomic status, lifestyle factors, and genetic susceptibility, may influence the observed racial differences in gastrointestinal cancer‐related mortality.^36^, ^37^ Additionally, racial and ethnic minorities are more likely to be diagnosed later in the disease, negatively impacting their prognosis due to reduced access to healthcare.^38^, ^39^, ^40^

A study by Bui *et al*. evaluated data on the five most common gastrointestinal cancers in the United States from 1 January 2013, to 31 December 2017, using the SEER registry.^41^ Colorectal cancer had the highest overall mortality (13.7%), followed by pancreatic (11.0%), liver (4.9%), esophageal (3.9%), and stomach cancer (3.0%). This is consistent with our results from 1999 to 2020 (Fig. 1). Bui *et al*. reported that non‐Hispanic Black populations had higher mortality rates from colorectal and pancreatic cancers. In comparison, non‐Asian/Pacific Islander and Hispanic individuals had lower mortality rates than non‐Hispanic Whites. Non‐American Indian and Alaskan Native individuals experienced lower mortality from pancreatic cancer than non‐Hispanic White individuals (MRR: 0.76 [95% CI 0.71–0.81]).

According to 2023 data from the American Cancer Society, the mortality rate for colorectal cancer among those diagnosed with colon cancer has declined from an annual rate of 4% to around 2% from 2012 to 2020.^42^ This trend is likely due to earlier detection and, thus, earlier cancer stages at diagnosis and improved treatments that have enhanced survival rates. Since 2005, the rate has been a 0.5% decline among those aged 50–64. Rates among Whites have held steady since 2014, while AI/AN individual have seen a 0.5% annual increase since 1990.^42^ Mortality rates for those under 50 have risen by 1% annually since 2004, notably among White, Hispanic, and AI/AN group. However, rates among Blacks have decreased since 1990. These data contributed to the recent move to lower the colorectal cancer screening age to 45 for earlier detection and treatment.^42^, ^43^ Our study reported age‐adjusted mortality rates instead of crude; therefore, their comparison between age groups would not have provided additional insight.

This study offers valuable insights into gastrointestinal cancer‐related mortality in the United States; however, certain limitations exist. The reliance on death certificates in the United States is flawed as death certificates are not issued by a physician at the time of death but by a physician who may not have seen the patient in months or years. This introduces inaccuracies less likely to occur when death certification requires physician assessment at the time of death, like in other countries. Furthermore, the International Classification of Diseases 10th Revision Clinical Modification codes may not capture all relevant cases. Additionally, despite a standard method, the Joinpoint regression analysis has inherent assumptions that could affect the accuracy of trend estimations. The study acknowledges the potential impact of the COVID‐19 pandemic on healthcare systems, cancer detection, treatment, and mortality rates in the later years. Data collection methods over time could influence observed trends. Lastly, the study does not account for other factors, such as socioeconomic status, lifestyle factors, and comorbidities, that may contribute to the observed differences in gastrointestinal cancer mortality rates and are unavailable in the CDC Wonder database.

In conclusion, our study demonstrates a significant decrease in overall gastrointestinal cancer mortality in the United States between 1999 and 2020. However, increasing mortality rates for liver and pancreatic cancers, likely related to the obesity epidemic, add to the importance of tackling the obesity epidemic. Overall, our findings contribute to a deeper understanding of GI cancer‐related mortality trends in the United States and offer valuable insights for developing targeted interventions and policies to reduce the burden of these cancers.

### Ethical statement

Institutional IRB approval was not obtained for this study as the CDC Wonder database is a third‐party de‐identified retrospective de‐identified database that is publicly accessible.

## Supporting information


**Appendix S1.** Trends of gastrointestinal cancers related mortality from 1999 to 2020 in the United States.

**Appendix S2.** Trends of gastrointestinal cancers related mortality from 1999 to 2020 in the United States stratified by sex.

**Appendix S3.** Trends of total gastrointestinal cancers related mortality from 1999 to 2020 in the United States stratified by race.
