# Supplementary material for: Trends in mortality from gastrointestinal, hepatic, and pancreatic cancers in the United States: A comprehensive analysis (1999–2020)
Source: JGH Open. 2024 Apr 15;8(4):e13064. doi: 10.1002/jgh3.13064 (PMC11017855; doi:10.1002/jgh3.13064)
Supplement: Supplementary file 1 — Appendix S1. Trends of gastrointestinal cancers related mortality from 1999 to 2020 in the United States. [file JGH3-8-e13064-s001.docx]

Supplementary Table 1: International classification of diseases (ICD) 10 codes used in the present study

| Location | ICD 10 codes |
| --- | --- |
| Esophagus | C15.x |
| Gastric | C16.x |
| Small bowel | C17.x |
| Colon | C18.x |
| Rectosigmoid | C19.x, C20.x |
| Anal | C21.x |
| Hepatic and intrahepatic biliary system | C22.x |
| Pancreatic | C25.x |
| Gallbladder | C23.x |
| Extrahepatic biliary system | C24.x |

**Trends in total gastrointestinal cancers related mortality from 1999 to 2020 in the United States**

**Esophageal cancers:**

Between 1999 and 2020, a total of 309919 deaths related to esophageal cancer, with an AAMR of 4.4 per 100,000 in 1999 to 3.7 per 100,000 in 2020 (AAPC -0.8% (95% CI: -1.0%, -0.6%, p < 0.001). There was no significant change in mortality rates from 1999-2006 (APC of -0.1% (95% CI: -0.6%, 0.4%, p= 0.582) followed by a significant decrease from 2006-2020 (APC of -1.2% (95% CI: -1.3%, -1.0%, p <0.001).

**Gastric cancers:**

Between 1999 and 2020, a total of 254105 deaths related to gastric cancer, with an AAMR of 4.6 per 100,000 in 1999 to 2.8 per 100,000 in 2020 (AAPC -2.5% (95% CI: -2.7% to -2.3%, p < 0.001). Between 1999 and 2008, mortality significantly declined, with an APC of -3.2% (95% CI: -3.6% to -2.9%, p < 0.001), followed by a slower decline from 2008 and 2020, with an APC of -2.0% (95% CI: -2.2% to -1.7%, p < 0.001)Between 1999 and 2020, a total of 28380 deaths related to small bowel cancer, with an AAMR of 0.4 per 100,000 in 1999 and 2020, with an AAPC of 0.2% (95% CI: -0.5% to 0.9%, p = 0.547).

**Colon cancers:**

Between 1999 and 2020, a total of 957232 deaths related to colon cancer, with an AAMR of 17.7 per 100,000 in 1999 to 9.8 per 100,000 in 2020 (AAPC -2.8% (95% CI: -3.0% to -2.7%, p < 0.001). Between 1999 and 2011, the mortality rates significantly declined, with an APC of -3.2% (95% CI: -3.4% to -3.0%, p < 0.001), followed by a slower decline from 2011 to 2020, with an APC of -2.4% (95% CI: -2.7% to -2.1%, p < 0.001).

**Rectal cancers:**

Between 1999 and 2020, a total of 208926 deaths related to rectal cancer, with an AAMR of 3.0 per 100,000 in 1999 to 2.8 per 100,000 in 2020 (AAPC -0.4% (95% CI: -0.7%, -0.1%, p=0.003). This trend was driven by a significant decrease in mortality rates from 1999-2015 (APC of -0.7% (95% CI: -0.9%, -0.5%, p<0.001)), followed by a non-significant increase in mortality rates from 2015-2020 (APC of 0.5% (95% CI: -0.7%, 1.6%, p=0.390)).

**Anal cancers:**

Between 1999 and 2020, a total of 18300 deaths related to anal cancer, with an AAMR of 0.2 per 100,000 in 1999 to 0.4 per 100,000 in 2020 (AAPC 2.8% (95% CI: 1.4%, 4.3%, p <0.001). From 1999 to 2010, the APC was -0.2% (95% CI: -2.1%, 1.9%, p= 0.873), followed by a significant increase in mortality from 2010 to 2020 with an APC of 6.2% (95% CI: 3.7%, 8.6%, p <0.001).

**Hepatic and intrahepatic cancers:**

Between 1999 and 2020, a total of 446959 deaths related to hepatic and intrahepatic cancers, with an AAMR of 4.5 per 100,000 in 1999 to 6.6 per 100,000 in 2020 (AAPC 1.8% (95% CI: 1.6%, 2.1%, p<0.001). From 1999-2007, the APC was 2.3% (95% CI: 2.0%, 2.6%, p<0.001) followed by APC of 3.1% (95% CI: 2.5%, 3.8%, p<0.001) from 2007-2013 and APC of 0.2% (95% CI: -0.2%, 0.6%, p=0.277) from 2013-2020.

**Pancreatic cancers**

Between 1999 and 2020, a total of 810628 deaths related to Pancreatic cancer, with an AAMR of 10.6 per 100,000 in 1999 to 11.1 per 100,000 in 2020 (AAPC of 0.2% (95% CI: 0.2%, 0.3%, p<0.001).

**Gallbladder cancers:**

Between 1999 and 2020, a total of 810628 deaths related to Gallbladder cancer, with an AAMR of 0.7 per 100,000 in 1999 to 0.5 per 100,000 in 2020 (AAPC of -1.4% (95% CI: -1.8%, -1.0%, p<0.001).

**Extrahepatic biliary tract cancers:**

Between 1999 and 2020, a total of 35324 deaths related to Extrahepatic biliary tract cancers, with an AAMR of 0.6 per 100,000 in 1999 to 0.5 per 100,000 in 2020 (AAPC of -0.8% (95% CI: -1.6%, 0.1%, p=0.091. The trend showed a significant decreasing trend from 1999-2011 (APC of -3.8%, 95% CI: -4.8%, -2.7%, p<0.001), followed by a significant increasing trend from 2011-2020 (APC of 3.4%, 95% CI: 1.7%, 5.2%, p=0.001).

**Trends in total gastrointestinal cancers related mortality from 1999 to 2020 in the United States stratified by gender**

**Esophageal cancers:**

Mortality rates for males remained stable from 1999 to 2006, with an APC of 0.2% (95% CI: -0.4%, 0.8%, p=0.470), followed by a significant decrease from 2006 to 2020, with an APC of -1.2% (95% CI: -1.4%, -1.0%, p<0.001) and an AAPC of -0.8% (95% CI: -1.0%, -0.6%, p<0.001). For females, there was a significant decrease in mortality rates from 1999 to 2020, with an AAPC of -1.4% (95% CI: -1.5%, -1.2%, p<0.001). Compared to males, females had a statistically significant lower AAPC, with an AAPC difference of -0.6% (95% CI: -0.9%, -0.3%, p<0.001), indicating a more pronounced decrease in mortality rates for females over the study period.

**Gastric cancers:**

Mortality rates declined significantly for both females and males over the study period. The average annual percent change (AAPC) for females was -2.3% (95% CI: -2.6% to -2.0%, p<0.001), and for males was -2.9% (95% CI: -3.0% to -2.7%, p<0.001). For females, there was a significant decline in mortality from 1999 to 2007, with an APC of -3.1% (95% CI: -3.7% to -2.4%, p<0.001), followed by a slower decline from 2007 to 2020, with an APC of -1.9% (95% CI: -2.2% to -1.6%, p<0.001). For males, there was a significant decline in mortality from 1999 to 2008, with an APC of -3.5% (95% CI: -3.8% to -3.2%, p<0.001), followed by a slower decline from 2008 to 2020, with an APC of -2.4% (95% CI: -2.6% to -2.2%, p<0.001). Compared to males, females had a statistically significant higher AAPC difference, with an AAPC difference of 0.5% (95% CI: 0.2% to 0.9%, p=0.001), indicating a more pronounced decline in mortality rates for males over the study period.

**Small bowel cancers:**

Mortality rates for females showed a non-significant increase over the entire study period (1999-2020), with an AAPC of 0.8% (95% CI: -0.1%, 1.8%, p=0.068). For males, there was a significant decline in mortality from 1999 to 2006, with an APC of -3.0% (95% CI: -6.6%, 0.8%, p=0.112), followed by a significant increase in mortality from 2006 to 2020, with an APC of 2.2% (95% CI: 0.8%, 3.6%, p=0.003) and an AAPC of 0.4% (95% CI: -1.0% to 1.9%, p=0.551). Compared to males, females had a statistically non-significant lower AAPC difference, with an AAPC difference of 0.4% (95% CI: -1.3%, 2.1%, p=0.632), indicating no significant difference in mortality rates compared to males over the study period.

**Colon cancers:**

Mortality rates for females showed a steep decline from 1999 to 2012 with an APC of -3.2% (95% CI: -3.4% to -3.1%, p<0.001), followed by a period of stability from 2012 to 2015 with an APC of -1.4% (95% CI: -4.8% to 2.0%, p=0.384), and then a slower decline from 2015 to 2020 with an APC of -2.9% (95% CI: -3.6% to -2.2%, p<0.001) and an AAPC of -2.9% (95% CI: -3.4% to -2.4%, p<0.001) for the study period. For males, there was a steep decline from 1999 to 2002 with an APC of -2.2% (95% CI: -3.5% to -0.9%, p=0.003), and from 2002 to 2005 with an APC of -4.6% (95% CI: -7.0% to -2.0%, p=0.002), followed by a slower decline from 2005 to 2012 with an APC of -3.0% (95% CI: -3.4% to -2.5%, p<0.001), and from 2012 to 2020 with an APC of -2.4% (95% CI: -2.7% to -2.2%, p<0.001) and an AAPC of -2.9% (95% CI: -3.3% to -2.5%, p<0.001) for the study period. Between 1999 and 2020, mortality rates declined significantly for both females and males, with an AAPC of -2.9% (95% CI: -3.4% to -2.4%, p<0.001) and -2.9% (95% CI: -3.3% to -2.5%, p<0.001), respectively. The AAPC difference between females and males was not statistically significant, with an AAPC difference of 0.0% (95% CI: -0.6% to 0.6%, p=0.987).

**Rectal cancers:**

Over the study period (1999-2020), female mortality rates decreased significantly with an average annual percent change (AAPC) of -0.6% (95% CI: -0.8%, -0.5%, p<0.001). For males, a significant decrease was observed from 1999-2015 (APC of -0.9% (95% CI: -1.1%, -0.7%, p<0.001)), followed by a non-significant increase from 2015-2020 (APC of 0.7% (95% CI: -0.5%, 2.0%, p=0.215)). When comparing the two cohorts, the difference in AAPC was not statistically significant (AAPC difference of -0.1% (95% CI: -0.5%, 0.2%, p=0.479)).

**Anal cancers:**

Over the study period (1999-2020), female mortality rates increased with an AAPC of 3.4% (95% CI: 2.6%, 4.2%, p <0.001. For males, there was a significant increasing trend in mortality, with an AAPC of 2.5% (95% CI: 1.0%, 4.1%, p = 0.002). When comparing the two cohorts, females had a higher AAPC than males (AAPC difference of 0.9%, 95% CI: -0.8%, 2.5%, p = 0.299).

**Hepatic/Intrahepatic biliary system cancer:**

For females (1999-2020), there was a significant increasing trend in mortality rates, with an AAPC of 1.6% (95% CI: 1.2%, 2.0%, p<0.001) while for males, an AAPC of 1.8% (95% CI: 1.6%, 2.0%, p<0.001) for the study period. In the females, from 1999-2008 ( APC 1.2% (95% CI: 0.8%, 1.6%, p<0.001), 2008-2013 (APC 3.6% (95% CI: 2.2%, 4.9%, p<0.001) and 2013-2020 (APC 0.7% (95% CI: 0.2%, 1.3%, p=0.013), there was a significant increasing trend of mortality. For males, there was a significant increasing trend from 1999-2014 (APC 2.7% (95% CI: 2.5%, 2.9%, p<0.001) and a non-significant decrease in mortality rates from 2014-2020 with an APC of -0.4% (95% CI: -1.0%, 0.3%, p=0.266). There was no significant AAPC difference among both cohorts ( 0.2%, 95% CI: -0.6%, 0.2%, p=0.384).

**Pancreatic cancer:**

The AAPC for females over the study period (1999-2020) was 0.2% (95% CI: 0.1%, 0.3%, p=0.002), while for males was 0.2% (95% CI: -0.0%, 0.4%, p=0.062). The trend for females was increasing from 1999-2008, with an APC of 0.4% (95% CI: 0.2%, 0.7%, p=0.001), followed by a non-significant trend from 2008-2020 (APC of 0.0%, 95% CI: -0.1%, 0.2%, p=0.932). Similarly, for males, here was a significant increasing trend in mortality rates from 1999-2017, with an APC of 0.3% (95% CI: 0.2%, 0.4%, p<0.001), followed by a non-significant trend from 2017-2020 (APC of -0.2%, 95% CI: -1.7%, 1.4%, p=0.820).There was no significant difference in AAPC among males and females (AAPC difference of -0.0%, 95% CI: -0.3%, 0.2%, p=0.891).

**Gallbladder cancer**

The AAPC for females over the study period (1999-2020) was -1.5% (95% CI: -1.9%, -1.1%, p<0.001), while for males was -1.2% (95% CI: -1.8%, -0.7%, p<0.001). There was no significant difference in AAPC between males and females (AAPC difference of -0.3%, 95% CI: -0.9%, 0.4%, p=0.431).

**Extrahepatic biliary system:**

The AAPC for females over the study period (1999-2020) was -1.6% (95% CI: -2.6%, -0.7%, p=0.001), while for males was -1.2% (95% CI: -2.4%, -0.0%, p=0.046). The trend for females showed a significant decrease from 1999-2003, with an APC of -8.2% (95% CI: -12.6%, -3.5%, p=0.002), followed by a non-significant change from 2003-2020 (APC of 0.0%, 95% CI: -0.5%, 0.5%, p=0.002). Similarly, for males, there was a significant decreasing trend in mortality rates from 1999-2008, with an APC of -4.7% (95% CI: -6.9%, -2.4%, p<0.001), followed by a non-significant change from 2008-2020 (APC of 1.4%, 95% CI: -0.1%, 3.0%, p=0.061). There was no significant difference in AAPC between males and females (AAPC difference of -0.4%, 95% CI: -1.9%, 1.2%, p=0.62).

**Trends in total gastrointestinal cancers related mortality from 1999 to 2020 in the United States stratified by race:**

**Esophageal cancers:**

For White individuals, there was a significant increase in mortality rates from 1999-2006, with an APC of 0.6% (95% CI: 0.1%, 1.2%, p = 0.033), followed by a significant decrease with an APC of -0.7% (95% CI: -0.9%, -0.5%, p <0.001) resulting in an AAPC of -0.2% (95% CI: -0.5%, 0.0%, p =0.027) for the whole study period. There was a significant decrease in mortality rates for Black or African American individuals during the entire study period (1999-2020), with an AAPC of -4.5% (95% CI: -4.7%, -4.3%, p <0.001). AA had an AAPC difference of -4.2% (95% CI: -4.5%, -4.0%, p <0.001), indicating a significant decrease in mortality rates compared to Whites over the study period. For Asian or Pacific Islander individuals, there was a significant decrease in mortality rates throughout the entire study period (1999-2020), with an AAPC of -1.3% (95% CI: -1.7%, -0.9%, p <0.001). Compared to Whites, AAPI had an AAPC difference of -1.1% (95% CI: -1.5%, -0.6%, p <0.001), indicating a significant decrease in mortality rates compared to Whites over the study period. For AI/AN, there was a significant decrease in mortality rates from 1999 to 2020, with an AAPC of -1.6% (95% CI: -2.5%, -0.6%, P = 0.002). Compared to Whites, AI/AN had an AAPC difference of -1.3% (95% CI: -2.2%, -0.4%, p = 0.004), indicating a significant decrease in mortality rates over the study period.

**Gastric cancers:**

From 1999 to 2006, the White population experienced a significant decline in mortality with an APC of -3.5% (95% CI: -4.2% to -2.8%, p < 0.001), followed by a slower decline from 2006 to 2020 with an APC of -2.0% (95% CI: -2.2% to -1.7%, p < 0.001), resulting in an AAPC of -2.5% (95% CI: -2.7% to -2.2%, p < 0.001) for the study period. Between 1999 and 2020, there was a significant decline in mortality for AA, with an AAPC of -3.3% (95% CI: -3.4% to -3.1%, p < 0.001). AA had an AAPC difference of -0.8% (95% CI: -1.1% to -0.5%, p < 0.001), indicating a significant decrease in mortality rates compared to Whites over the study period. Between 1999 and 2020, there was a significant decline in mortality among the AAPI, with an AAPC of -3.7% (95% CI: -4.0% to -3.5%, p < 0.001). When comparing the mortality trends of AAPI to White individuals, there was an AAPC difference of -1.2% (95% CI: -1.6% to -0.9%, p < 0.001), indicating a significant decrease in mortality rates among AAPI compared to White individuals over the study period. Between 1999 and 2020, the mortality rates for AI/AN declined significantly, with an APC of -3.2% (95% CI: -3.9% to -2.5%, p < 0.001). However, there was no significant difference in AAPC between AI/AN and Whites (AAPC difference = -0.7%, 95% CI: -1.4% to 0.0%, p = 0.060).

**Small bowel cancers:**

The mortality rates for White individuals declined significantly from 1999 to 2000 with an APC of 2.3% (95% CI: 0.6% to 4.1%, p = 0.011), followed by a significant increase from 2006 to 2020 with an APC of 2.3% (95% CI: 0.6% to 4.1%, p = 0.011) but a nonsignificant AAPC of 0.2% (95% CI: -1.6% to 2.1%, p = 0.802) for the study period. For AA, there was a significant increase in mortality rates from 1999 to 2020, with an APC of 1.1% (95% CI: 0.3% to 1.8%, p = 0.008). AA had an AAPC difference of 0.8% (95% CI: -1.1% to 2.8%, p = 0.413), indicating no difference in mortality rates compared to Whites over the study period. Additional races were not included due to fewer deaths.

**Colon cancers:**

The mortality rates for whites declined significantly from 1999 to 2011, with an APC of -3.2% (95% CI: -3.4% to -3.1%, p < 0.001) and from 2011 to 2020, with an APC of -2.2% (95% CI: -2.5% to -1.9%, p < 0.001) resulting in AAPC of -2.8% (95% CI: -3.0% to -2.6%, p < 0.001) for the study period. Between 1999 and 2020, the mortality rates for AA declined significantly, with an AAPC of -3.2% (95% CI: -3.3% to -3.0%, p < 0.001). AA had an AAPC difference of -0.4% (95% CI: -0.5% to -0.2%, p < 0.001), indicating decreased mortality rates compared to Whites over the study period. AAPI mortality rates increased between 1999 and 2001 (APC of 4.4%, 95% CI: -5.7% to 15.5%, p = 0.382), followed by a significant decline from 2001 to 2020 with an APC of -2.4% (95% CI: -2.7% to -2.1%, p < 0.001) resulting in AAPC of -1.8% (95% CI: -2.7% to -0.9%, p < 0.001) for the study period. Compared to whites, AAPI had an AAPC difference of 1.0% (95% CI: 0.1% to 1.9%, p = 0.037) in mortality rates between 1999 and 2020, indicating an increase in mortality rates compared to Whites over the study period. Between 1999 and 2020, the mortality rates for AI/AN declined significantly, with an AAPC of -1.9% (95% CI: -2.4% to -1.3%, p < 0.001). Compared to whites, AI/AN had an AAPC difference of 0.9% (95% CI: 0.4% to 1.5%, p = 0.001), indicating that the mortality rates for AI/AN declined at a slower rate compared to the mortality rates for whites for the study period.

**Rectal cancers:**

The AAPC for AA over the entire study period (1999-2020) was -0.6% (95% CI: -1.2%, 0.0%, p=0.065), while for Whites, it was -0.5% (95% CI: -0.6%, -0.3%, p<0.001). There was no significant change in mortality rates for AA from 1999 to 2010, with an APC of -0.1% (95% CI: -0.5%, 0.3%, p=0.629). However, from 2010 to 2014, there was a steep decline, with an APC of -3.5% (95% CI: -6.5%, -0.4%, p=0.028), followed by a non-significant increase from 2014 to 2020, with an APC of 0.4% (95% CI: -0.6%, 1.5%, p=0.390). The AAPC difference between AA and Whites was not statistically significant, with an AAPC difference of -0.1% (95% CI: -0.8%, 0.5%, p=0.704), indicating no significant difference in mortality rates between the two groups over the study period. The AAPC for Asian or Pacific Islander individuals over the entire study period (1999-2020) was -0.5% (95% CI: -0.9%, -0.2%, p=0.003). No significant difference was found between the AAPC of AAPI compared to whites, with an AAPC difference of -0.1% (95% CI: -0.4%, 0.3%, p=0.715). The AAPC for AI/AN individuals over the entire study period (1999-2020) was -0.9% (95% CI: -2.1%, 0.3%, p=0.126), indicating a non-significant decline in mortality rates. No significant difference was found between the AI/AN compared to whites with an AAPC difference of -0.4% (95% CI: -1.6%, 0.7%, p=0.449).

**Anal cancers:**

There was a non-significant increase in mortality rates for AA, with an AAPC of 2.7% (95% CI: -0.3%, 5.8%, p=0.077), while for whites, there was a significant increase in mortality rates, with an AAPC of 3.2% (95% CI: 1.9%, 4.5%, p<0.001) over the study period. For AA, from 1999-2007, there was a non-significant change in mortality rates, with an APCof -0.1% (95% CI: -2.4%, 2.2%, p=0.895), while from 2007-2010 (APC 13.2% (95% CI: -8.4%, 39.9%, p=0.231) and 2010-2020 (APC 2.0% (95% CI: 0.4%, 3.7%, p=0.020) there was a significant increase in mortality. For the Whites, there was a non-significant change in mortality rates from 1999-2008 (APC -0.2% (95% CI: -2.6%, 2.3%, p=0.860), followed by a significant increase from 2008-2020 (APC 5.8% (95% CI: 4.2%, 7.5%, p<0.001).Whites had a higher AAPC than AA (AAPC difference of -0.5%, 95% CI: -3.8%, 2.8%, p=0.774). Additional races were not compared due to less number of cases.

**Hepatic/Intrahepatic biliary system cancer:**

Over the full study period (1999-2020), there was a significant increasing trend in mortality rates for AA (AAPC of 1.2% (95% CI: 0.6%, 1.7%, p<0.001) and whites (AAPC of 2.1% (95% CI: 1.9%, 2.3%, p<0.001). There was a significant increasing trend for AA from 1999-2013 (APC of 2.6% (95% CI: 2.4%, 2.8%, p<0.001), with a nonsignificant change from 2013-2017 (APC of 0.3% (95% CI: -2.2%, 2.9%, p=0.788), followed by a significant decreasing trend from 2017-2020 (APC of -4.1% (95% CI: -6.5%, -1.7%, p=0.003). For Whites, there was a significant increasing trend from 1999-2015 (APC of 2.7% (95% CI: 2.6%, 2.8%, p<0.001), followed by a non-significant change in mortality rates, with an APC of 0.0% (95% CI: -0.8%, 0.8%, p=0.984) from 2015-2020. Whites had a higher AAPC than AA (AAPC difference of -0.9%, 95% CI: -1.5%, -0.3%, p=0.004). For the study period, there was a significant decreasing trend in mortality rates for the AAPI, with an AAPC of -1.5% (95% CI: -2.0%, -0.9%, p<0.001) with two segments 1999-2013 (APC of -0.8% (95% CI: -1.3%, -0.3%, p=0.006) and 2013-2020 (APC of -2.8% (95% CI: -4.3%, -1.4%, p=0.001). Compared to whites, AAPI had lower mortality (AAPC difference of -3.5%, 95% CI: -4.1%, -2.9%, p<0.001). From 1999-2020, there was a significant increasing trend in mortality rates for the AI/AN, with an AAPC of 1.3% (95% CI: 0.6%, 1.9%, p=0.001) and a lower AAPC than whites (AAPC difference of -0.8%, 95% CI: -1.4%, -0.1%, p=0.016).

**Pancreatic cancer:**

The mortality rates for AA decreased over the study period (1999-2020) with an AAPC of -0.3% (95% CI: -0.4%, -0.2%, p<0.001), while for whites, mortality increased by an AAPC of 0.3% (95% CI: 0.3%, 0.4%, p<0.001). AA had lower mortality than whites (AAPC difference of -0.6%, 95% CI: -0.7%, -0.5%, p<0.001). The mortality rates for AAPI had no change over the study period (1999-2020) with an AAPC of -0.0% (95% CI: -0.3%, 0.3%, p = 0.973). AAPI had lower mortality than whites (AAPC difference of -0.3%, 95% CI: -0.7%, 0%, p = 0.05). The mortality rates for AI/AN had no change over time, with an AAPC of -0.1% (95% CI: -0.7%, 0.6%, p = 0.771). AI/AN had no difference in mortality than whites (AAPC difference of -0.4%, 95% CI: -0.1%, 0.2%, p = 0.18).

**Gallbladder cancer**

For AA, there was no significant trend in mortality rates over the entire study period (1999-2020) with an AAPC of 0.1% (95% CI: -0.6%, 0.8%, p=0.764). For the White population, there was a significant decreasing trend from 1999-2003 (APC of -4.4% (95% CI: -8.5%, -0.2%, p=0.041), followed by a non-significant trend from 2003-2012 (APC of -0.1% (95% CI: -1.6%, 1.4%, p=0.883) and a significant decreasing trend from 2012-2020 (APC of -2.8% (95% CI: -4.2%, -1.3%, p=0.001) (AAPC -2.0% 95%CI -3.0%, -0.9%, p < 0.001). Compared to white, AA had higher mortality for the study period (AAPC difference of 2.1%, 95% CI: 0.8%, 3.3%, p=0.001). The AAPC for AAPI over the study period (1999-2020) was -1.2% (95% CI: -2.2%, -0.1%, p=0.031) with no significant difference from whites (AAPC difference of 0.4%, 95% CI: -0.8%, 1.6%, p=0.532). AI/AN were not included due to less number of cases.

**Extrahepatic biliary system:**

The AAPC for AA over the study period (1999-2020) was 0.4% (95% CI: -0.6%, 1.3%, p=0.439), while for Whites was -0.5% (95% CI: -1.6%, 0.7%, p=0.391). For Whites, there was a significant decreasing trend from 1999-2013, with an APC of -2.9% (95% CI: -3.9%, -1.8%, p<0.001), followed by a significant increasing trend from 2013-2020 (APC of 4.5%, 95% CI: 1.3%, 7.7%, p=0.008). A significant difference in AAPC between AA and whites was present (AAPC difference of 0.9%, 95% CI: -0.6%, 2.3%, p=0.245). The AAPC for AAPI over the study period (1999-2020) was -1.0% (95% CI: -2.0%, -0.0%, p=0.049) with no significant difference in AAPC compared to whites (AAPC difference of -0.5%, 95% CI: -2.0%, 1.0%, p=0.507). AI/AN were not included due to less number of cases.

**Trends in total gastrointestinal cancers related mortality from 1999 to 2020 in the United States stratified by age group**

**Total cancers:**

For total cancers of the GI tract, the 25-44 age group experienced a slight decrease in mortality with an AAPC of -0.3%* (95% CI: -0.5 to -0.1, p = 0.002) over the study period from 1999-2020. In contrast, the 45-64 age group experienced a slight increase in mortality with an AAPC of 0.3%* (95% CI: 0.1 to 0.4, p < 0.001). Comparing the two age groups showed that the 25-44 age group experienced a significantly more significant decrease in mortality with an AAPC difference of -0.6%* (95% CI: -0.9 to -0.4, p < 0.001). For the 65+ age group, there was a significant decrease in mortality with an AAPC of -1.3%* (95% CI: -1.5 to -1.2, p < 0.001) and an AAPC difference of 1.0%* (95% CI: 0.7 to 1.2, p < 0.001) compared to the age group 25-44 years.

**Colon cancers:**

For colon cancer, the 25-44 age group experienced a slight decrease in mortality rates with an AAPC of -0.3% (95% CI: -1.8 to 1.2,p=0.694). In contrast, the 45-64 age group had a significant decrease in mortality rates, with an AAPC of -1.1%* (95% CI: -1.3 to -0.8, period (p<0.001). In comparison, there was no significant difference in AAPC (0.8, 95% CI: -0.7 to 2.2; p=0.318). For the 65+ age group, there was a significant decrease in mortality rates, with an AAPC of -3.6%* (95% CI: -3.8 to -3.4; p<0.001), and a significantly greater decrease compared to the 25-44 age group with an AAPC difference of 3.3%* (95% CI: 1.8 to 4.8; p<0.001).

**Hepatic/Intrahepatic biliary tract cancers:**

For liver and intrahepatic biliary tract cancers, the 25-44 age group had a significant decrease in mortality rates with an AAPC of -1.1%* (95% CI: -1.7 to -0.4, p=0.001), while the 45-64 age group had a significant increase in mortality rates with an AAPC of 2.5%* (95% CI: 1.8 to 3.2,p<0.001) over the study period. Comparing the two age groups showed a significant difference in AAPC of -3.6%* (95% CI: -4.5 to -2.6; p<0.001). For the 65+ age group, there was a significant increase in mortality rates with an AAPC of 2.0%* (95% CI: 1.8 to 2.2,p<0.001) and a significantly greater increase compared to the 25-44 age group with an AAPC difference of -3.1%* (95% CI: -3.8 to -2.4; p<0.001)

**Pancreatic cancers:**

For pancreatic cancers, the 25-44 age group had a significant decrease in mortality rates with an AAPC of -1.4% (95% CI: -1.8 to -1.0, p<0.001), while the 45-64 age group had a significant increase in mortality rates with an AAPC of 0.8% (95% CI: 0.4 to 1.2, p<0.001) over the study period. Comparing the two age groups showed a significant AAPC difference of -2.2% (95% CI: -2.7 to -1.6; p<0.001). For the 65+ age group, there was no significant change in mortality rates with an AAPC of 0.1 (95% CI: -0.0 to 0.2; p=0.097) and no significant difference in AAPC compared to the 25-44 age group(-1.4%, 95% CI: -2.3 to -0.4; p=0.003).
