# Supplementary material for: Trends in mortality from gastrointestinal, hepatic, and pancreatic cancers in the United States: A comprehensive analysis (1999–2020)
Source: JGH Open. 2024 Apr 15;8(4):e13064. doi: 10.1002/jgh3.13064 (PMC11017855; doi:10.1002/jgh3.13064)
Supplement: Supplementary file 2 — Appendix S2. Trends of gastrointestinal cancers related mortality from 1999 to 2020 in the United States stratified by sex. [file JGH3-8-e13064-s003.pdf]

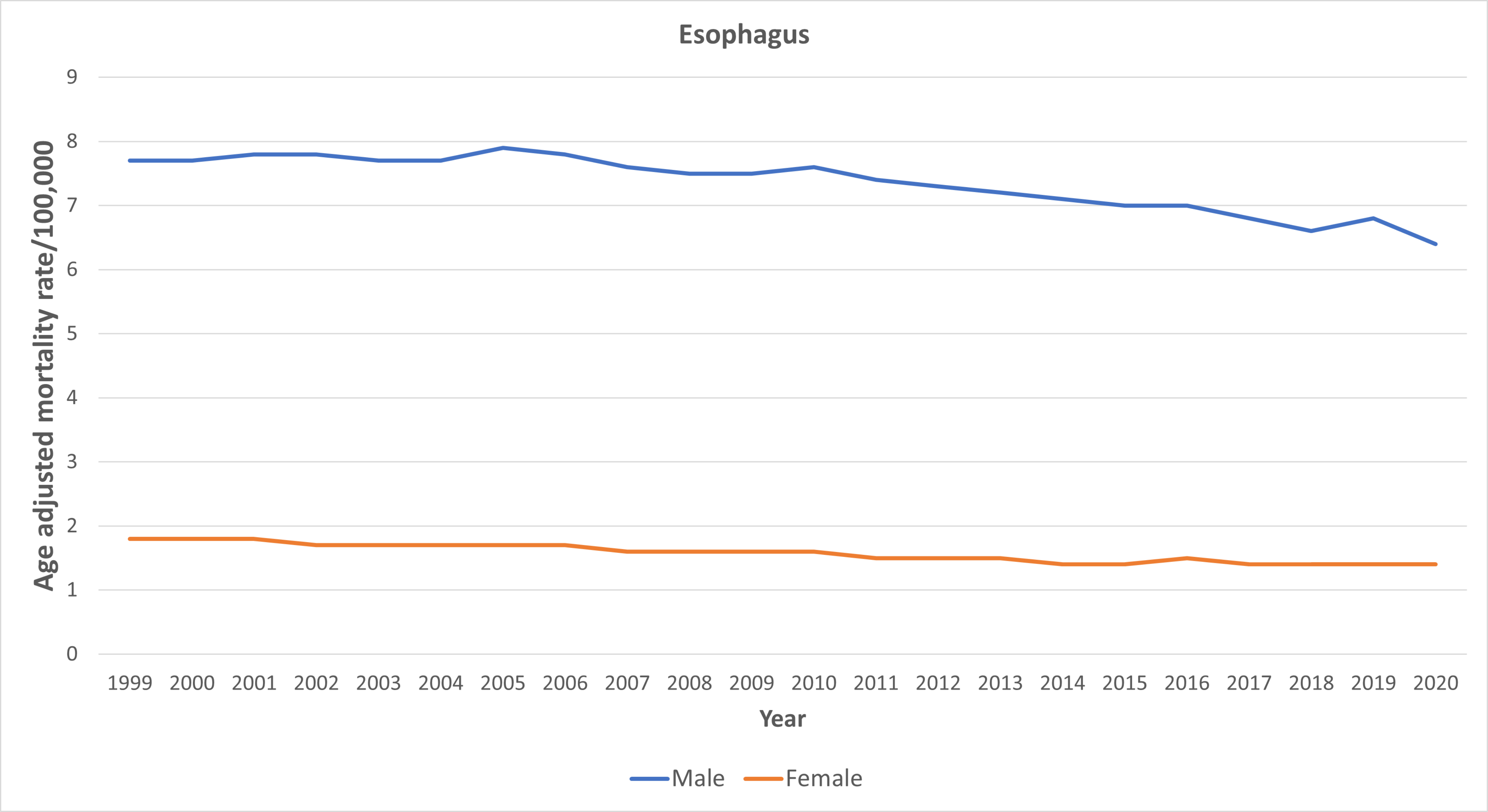
Esophagus
Age adjusted mortality rate/100,000
9
8
7
6
5
4
3
2
1
0
1999
2000
2001
2002
2003
2004
2005
2006
2007
2008
2009
2010
2011
2012
2013
2014
2015
2016
2017
2018
2019
2020
Year
Male
Female

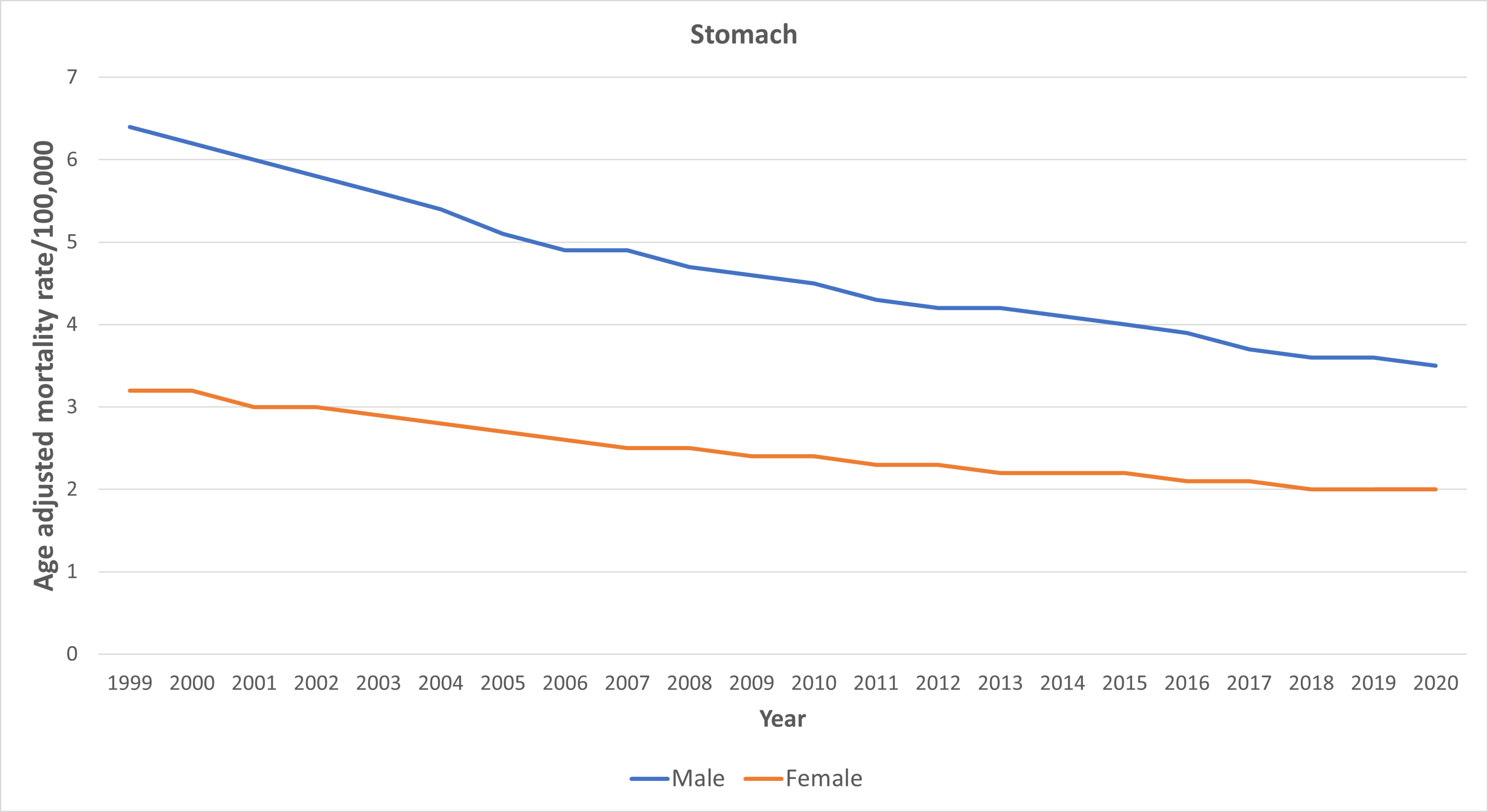
Stomach
Age adjusted mortality rate/100,000
7
6
5
4
3
2
1
0
1999
2000
2001
2002
2003
2004
2005
2006
2007
2008
2009
2010
2011
2012
2013
2014
2015
2016
2017
2018
2019
2020
Year
Male
Female

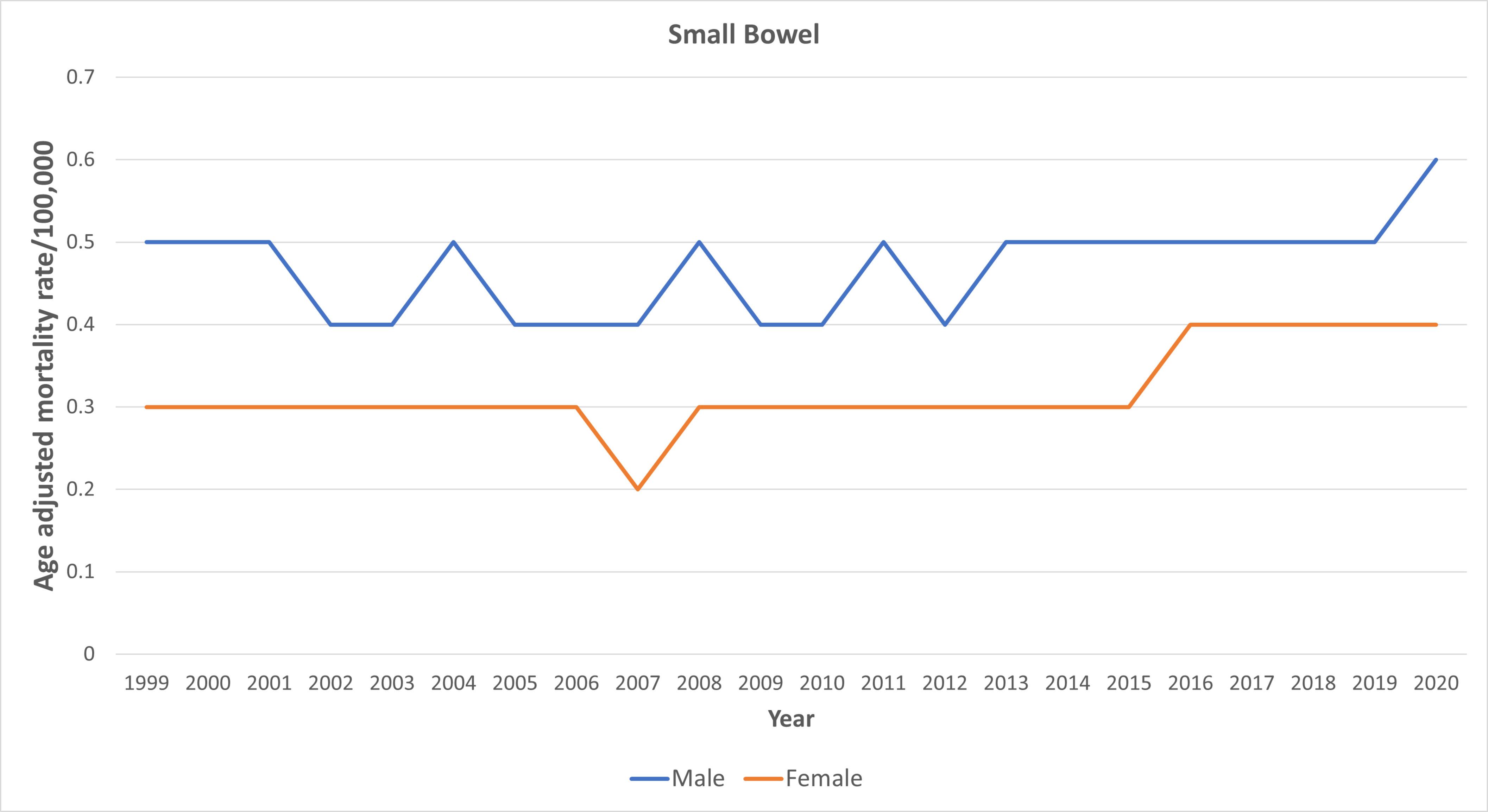

Small Bowel
Age adjusted mortality rate/100,000
0.7
0.6
0.5
0.4
0.3
0.2
0.1
0
1999 2000 2001 2002 2003 2004 2005 2006 2007 2008 2009 2010 2011 2012 2013 2014 2015 2016 2017 2018 2019 2020
Year
Male
Female

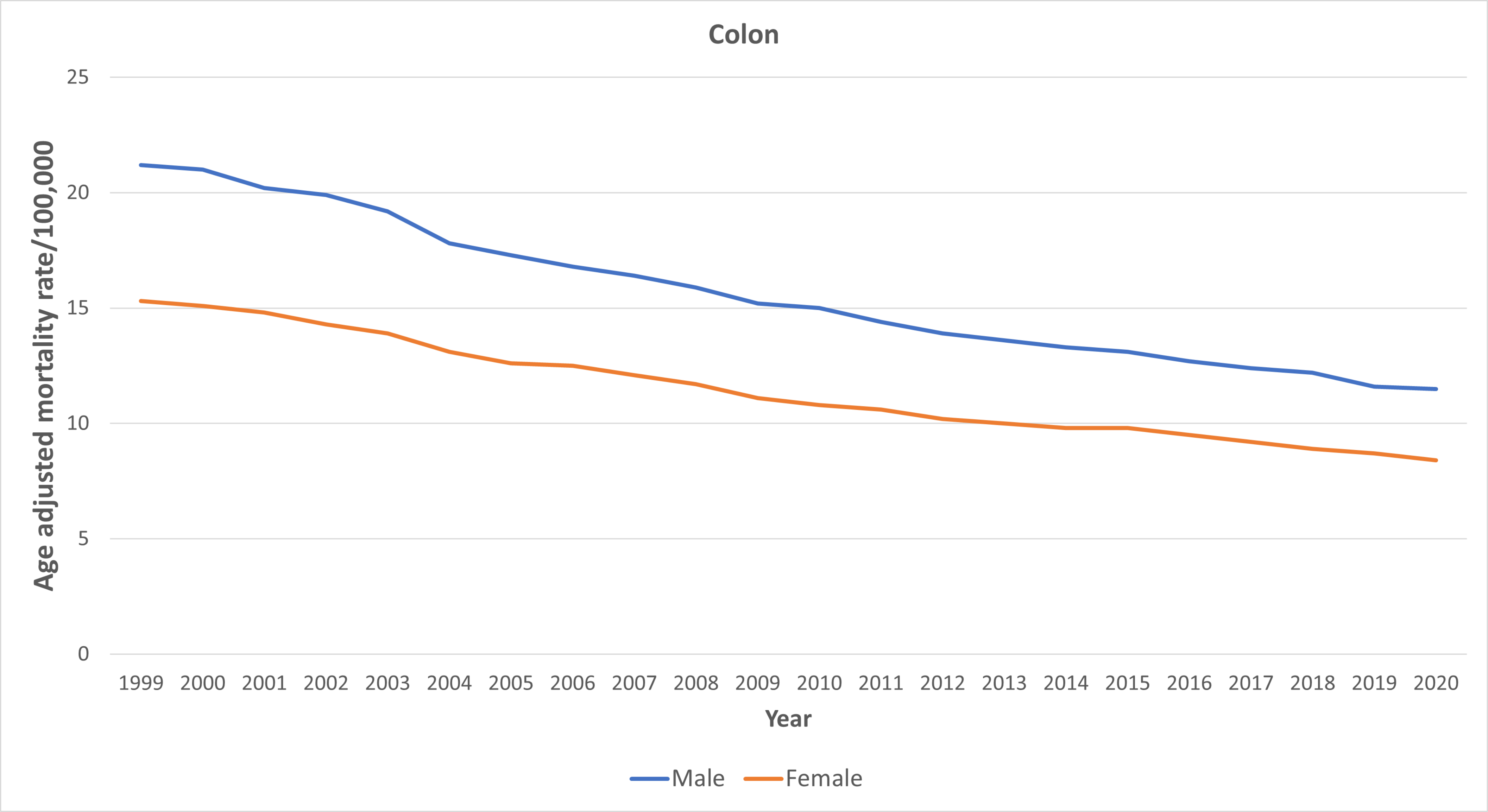

Colon
Age adjusted mortality rate/100,000
25
20
15
10
5
0
1999 2000 2001 2002 2003 2004 2005 2006 2007 2008 2009 2010 2011 2012 2013 2014 2015 2016 2017 2018 2019 2020
Year
Male
Female

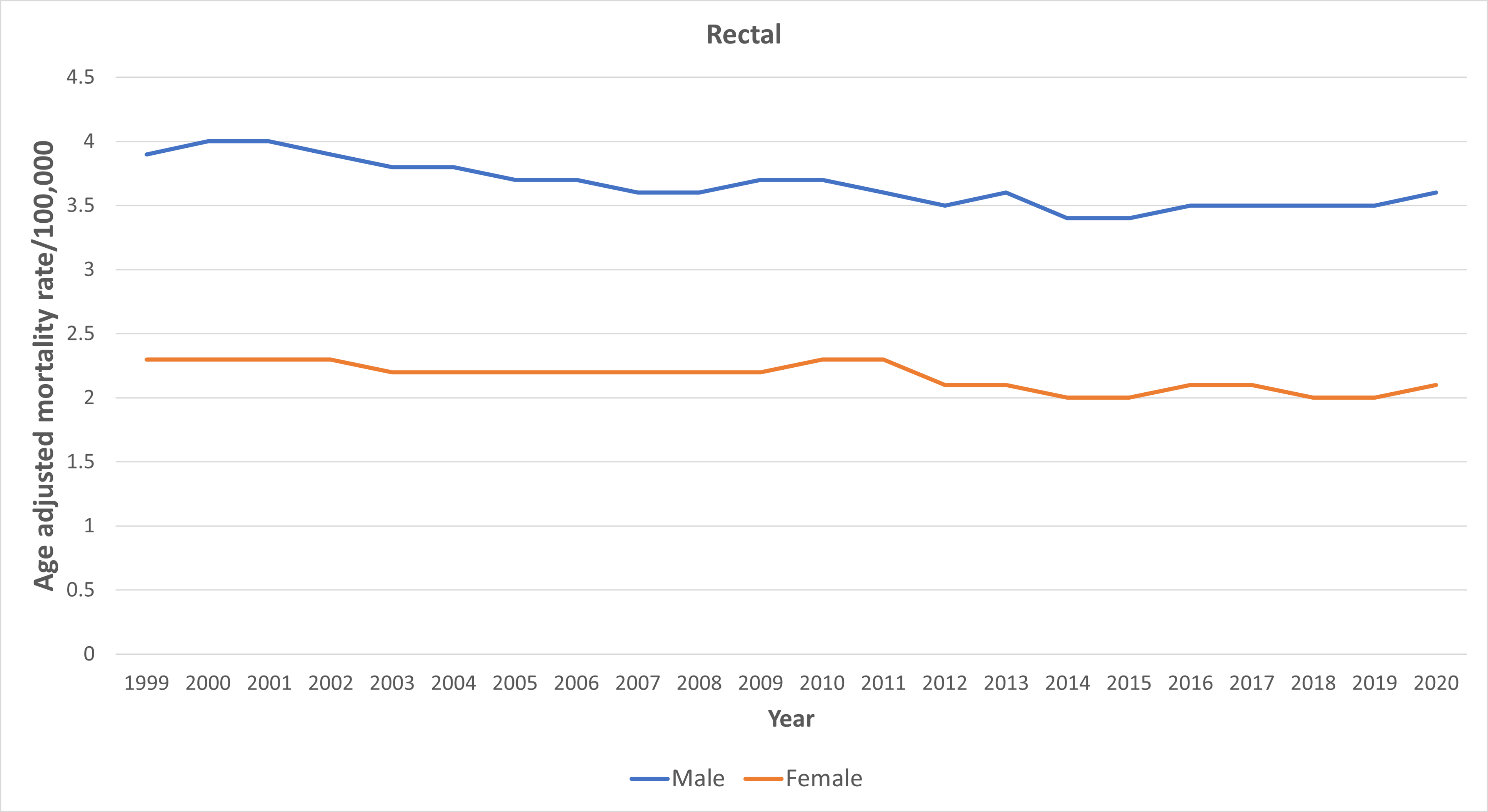
Rectal
Age adjusted mortality rate/100,000
4.5
4
3.5
3
2.5
2
1.5
1
0.5
0
1999
2000
2001
2002
2003
2004
2005
2006
2007
2008
2009
2010
2011
2012
2013
2014
2015
2016
2017
2018
2019
2020
Year
Male
Female

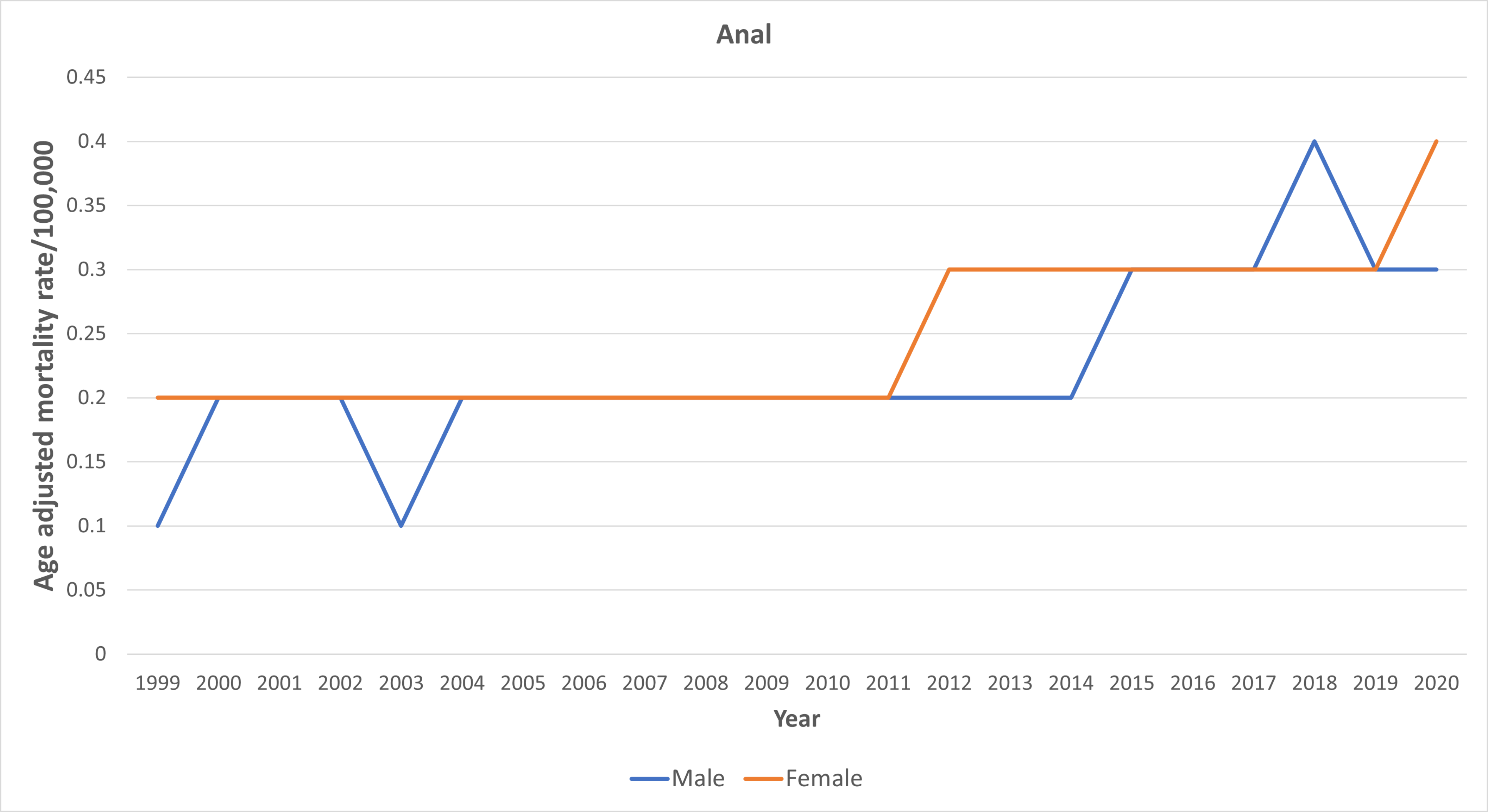
Anal
Age adjusted mortality rate/100,000
0.45
0.4
0.35
0.3
0.25
0.2
0.15
0.1
0.05
0
1999 2000 2001 2002 2003 2004 2005 2006 2007 2008 2009 2010 2011 2012 2013 2014 2015 2016 2017 2018 2019 2020
Year
Male
Female

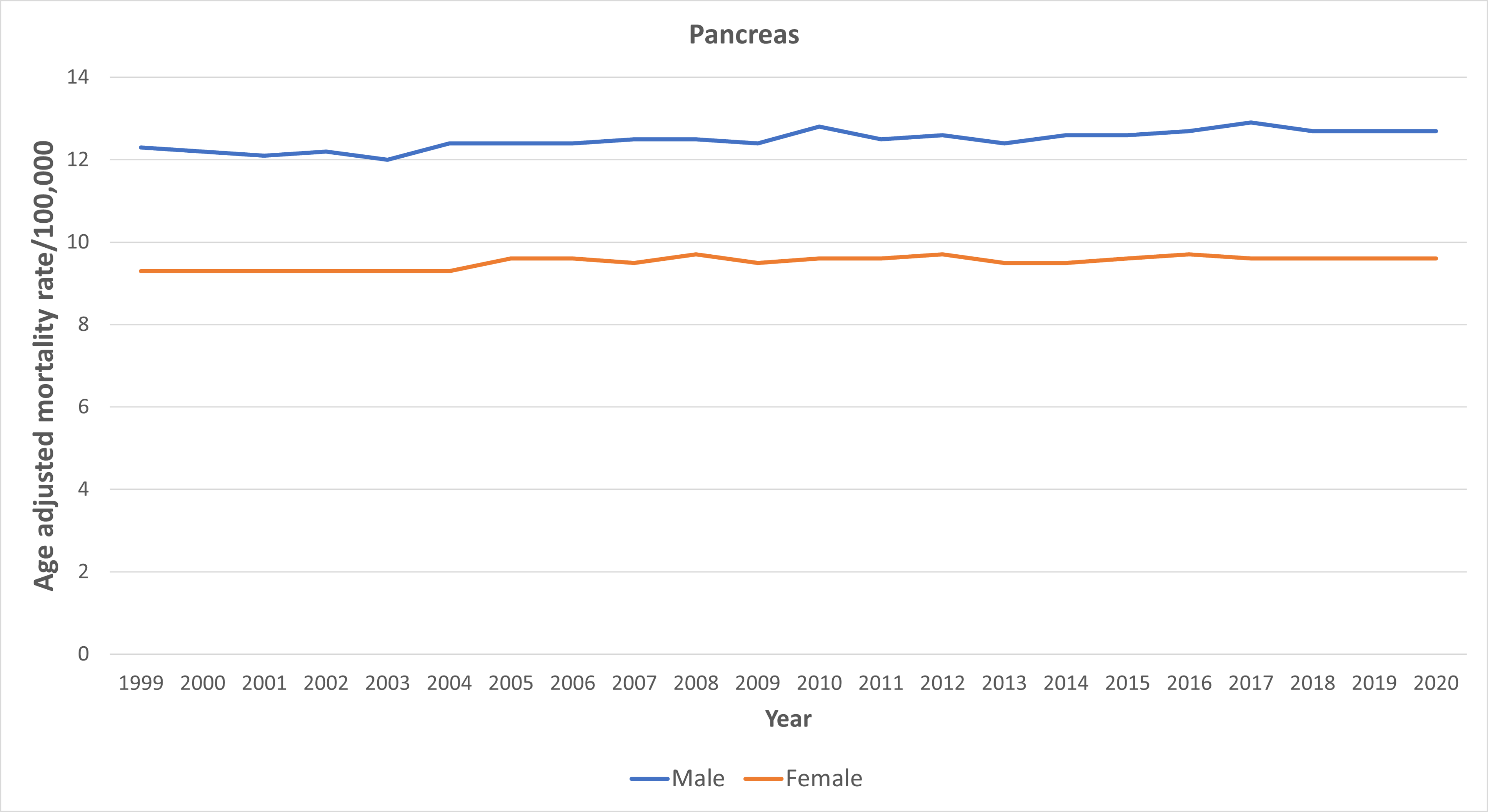

Pancreas
Age adjusted mortality rate/100,000
14
12
10
8
6
4
2
0
1999
2000
2001
2002
2003
2004
2005
2006
2007
2008
2009
2010
2011
2012
2013
2014
2015
2016
2017
2018
2019
2020
Year
Male
Female

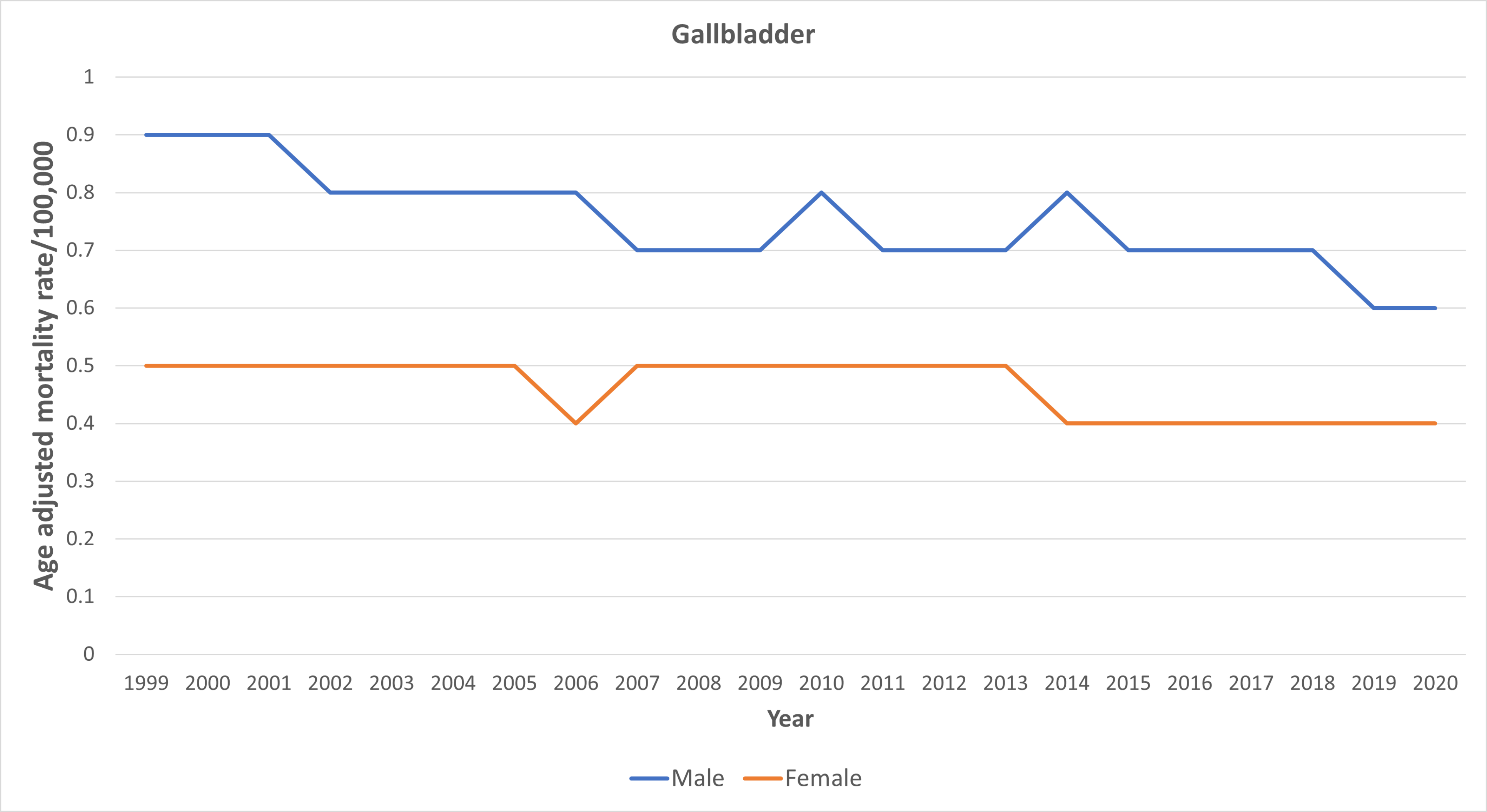
Gallbladder
Age adjusted mortality rate/100,000
1
0.9
0.8
0.7
0.6
0.5
0.4
0.3
0.2
0.1
0
1999
2000
2001
2002
2003
2004
2005
2006
2007
2008
2009
2010
2011
2012
2013
2014
2015
2016
2017
2018
2019
2020
Year
Male
Female

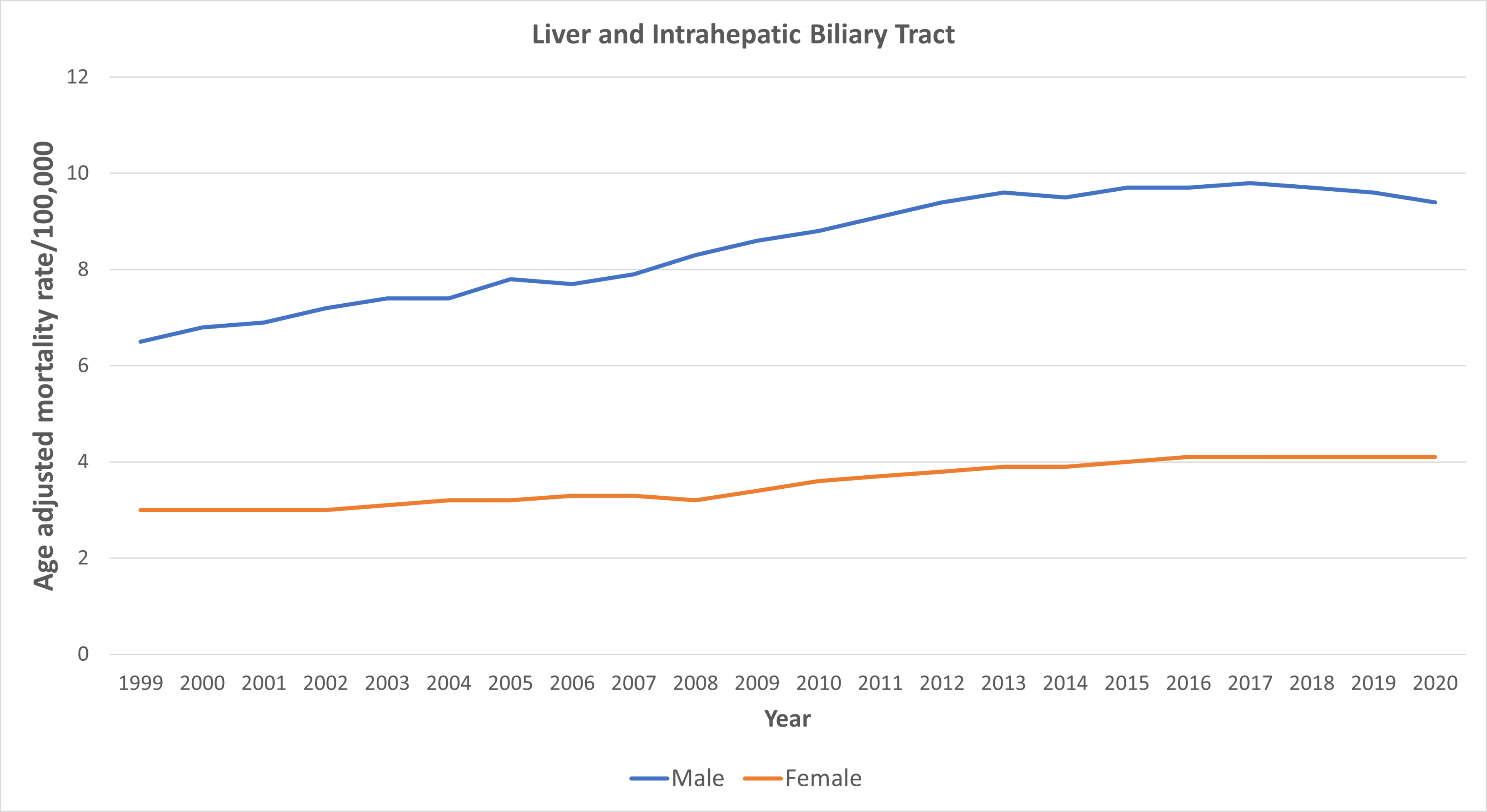

Liver and Intrahepatic Biliary Tract
Age adjusted mortality rate/100,000
0
2
4
6
8
10
12
1999
2000
2001
2002
2003
2004
2005
2006
2007
2008
2009
2010
2011
2012
2013
2014
2015
2016
2017
2018
2019
2020
Year
Male
Female

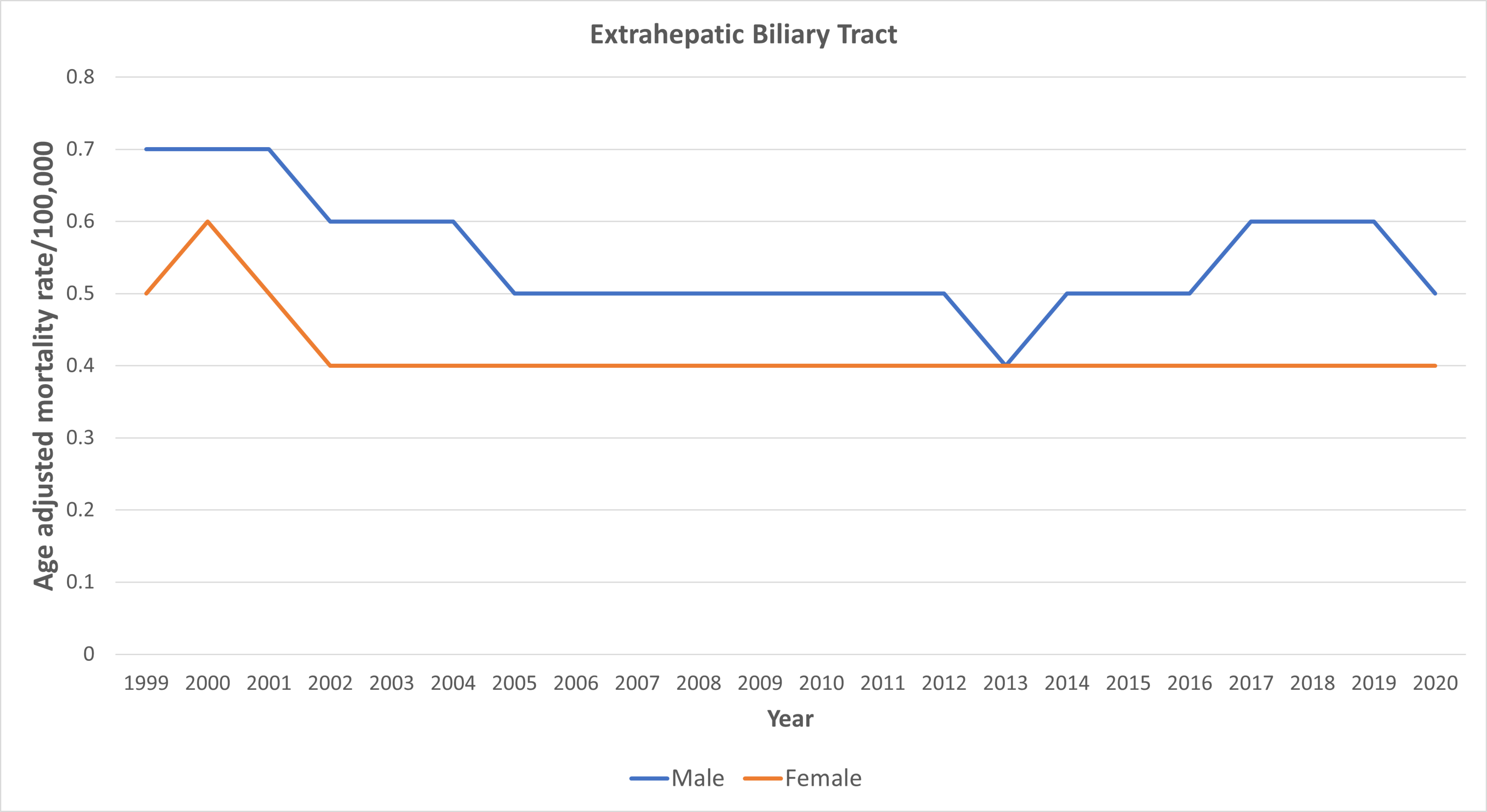
Extrahepatic Biliary Tract
0.8
0.7
0.6
0.5
0.4
0.3
0.2
0.1
0
Age adjusted mortality rate/100,000
1999 2000 2001 2002 2003 2004 2005 2006 2007 2008 2009 2010 2011 2012 2013 2014 2015 2016 2017 2018 2019 2020
Year
Male
Female
