# Supplementary material for: Trends in mortality from gastrointestinal, hepatic, and pancreatic cancers in the United States: A comprehensive analysis (1999–2020)
Source: JGH Open. 2024 Apr 15;8(4):e13064. doi: 10.1002/jgh3.13064 (PMC11017855; doi:10.1002/jgh3.13064)
Supplement: Supplementary file 3 — Appendix S3. Trends of total gastrointestinal cancers related mortality from 1999 to 2020 in the United States stratified by race. [file JGH3-8-e13064-s002.pdf]

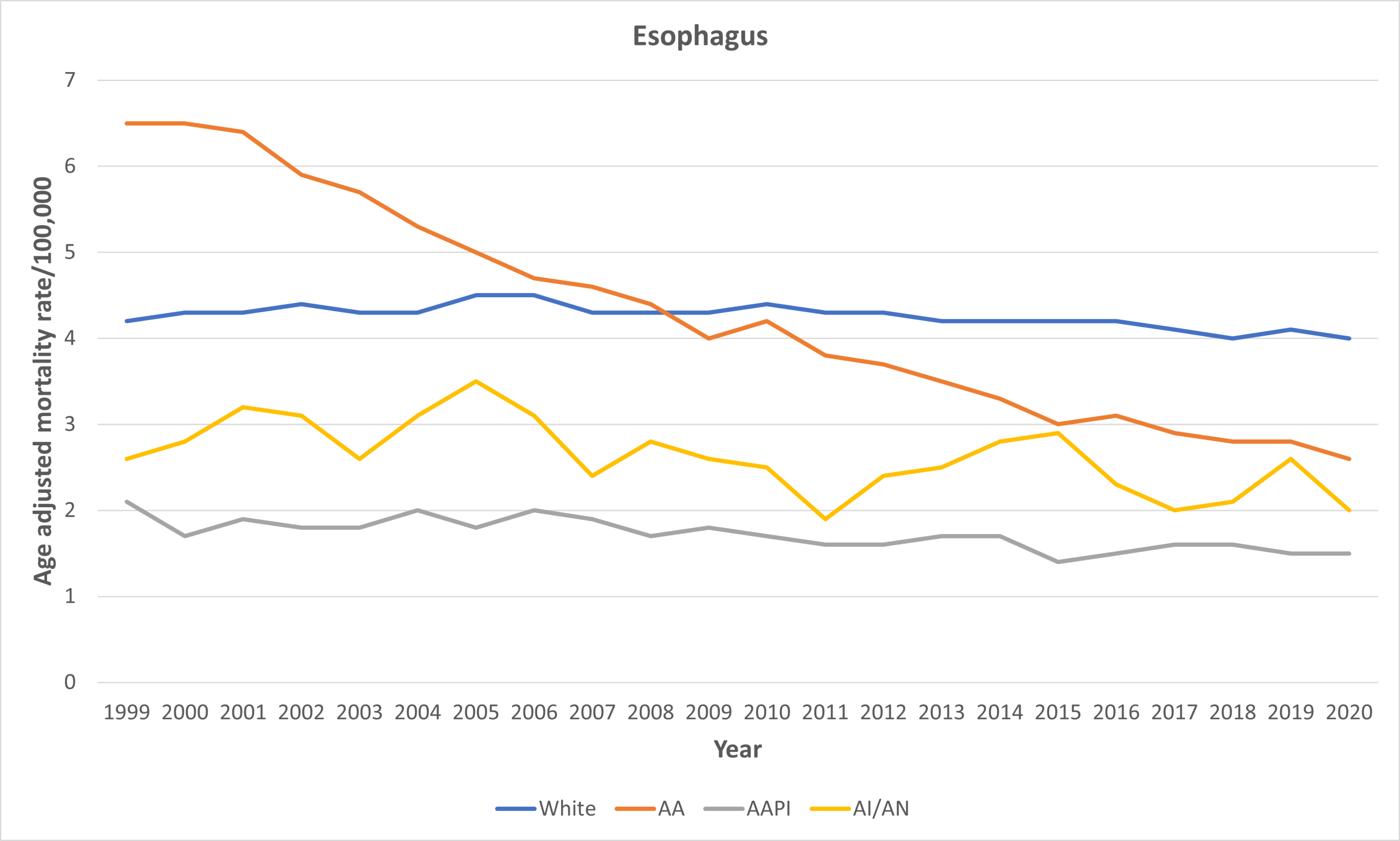
Esophagus
Age adjusted mortality rate/100,000
0
1
2
3
4
5
6
7
1999 2000 2001 2002 2003 2004 2005 2006 2007 2008 2009 2010 2011 2012 2013 2014 2015 2016 2017 2018 2019 2020
Year
White
AA
AAPI
AI/AN

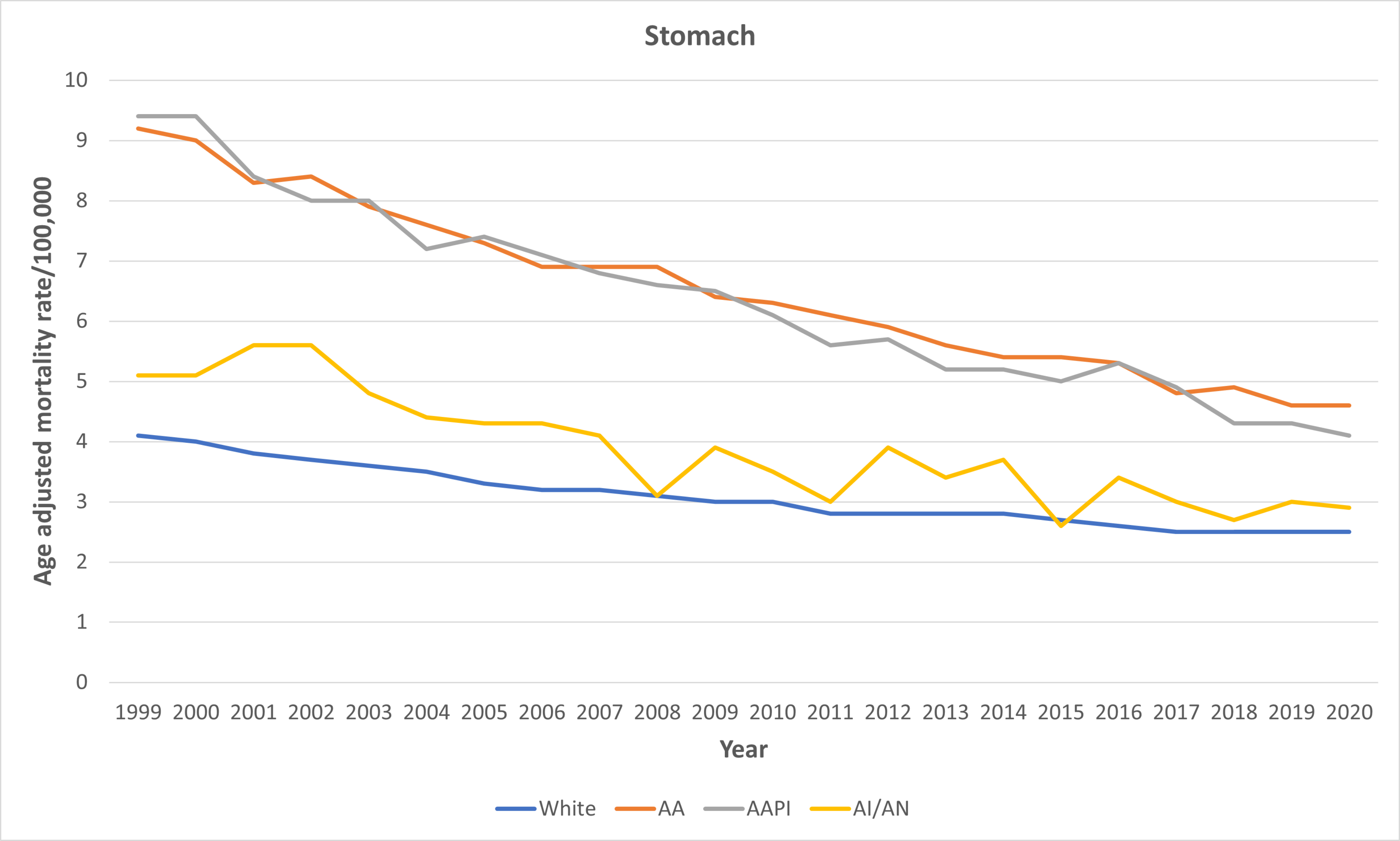

Stomach
Age adjusted mortality rate/100,000
10
9
8
7
6
5
4
3
2
1
0
1999 2000 2001 2002 2003 2004 2005 2006 2007 2008 2009 2010 2011 2012 2013 2014 2015 2016 2017 2018 2019 2020
Year
White
AA
AAPI
AI/AN

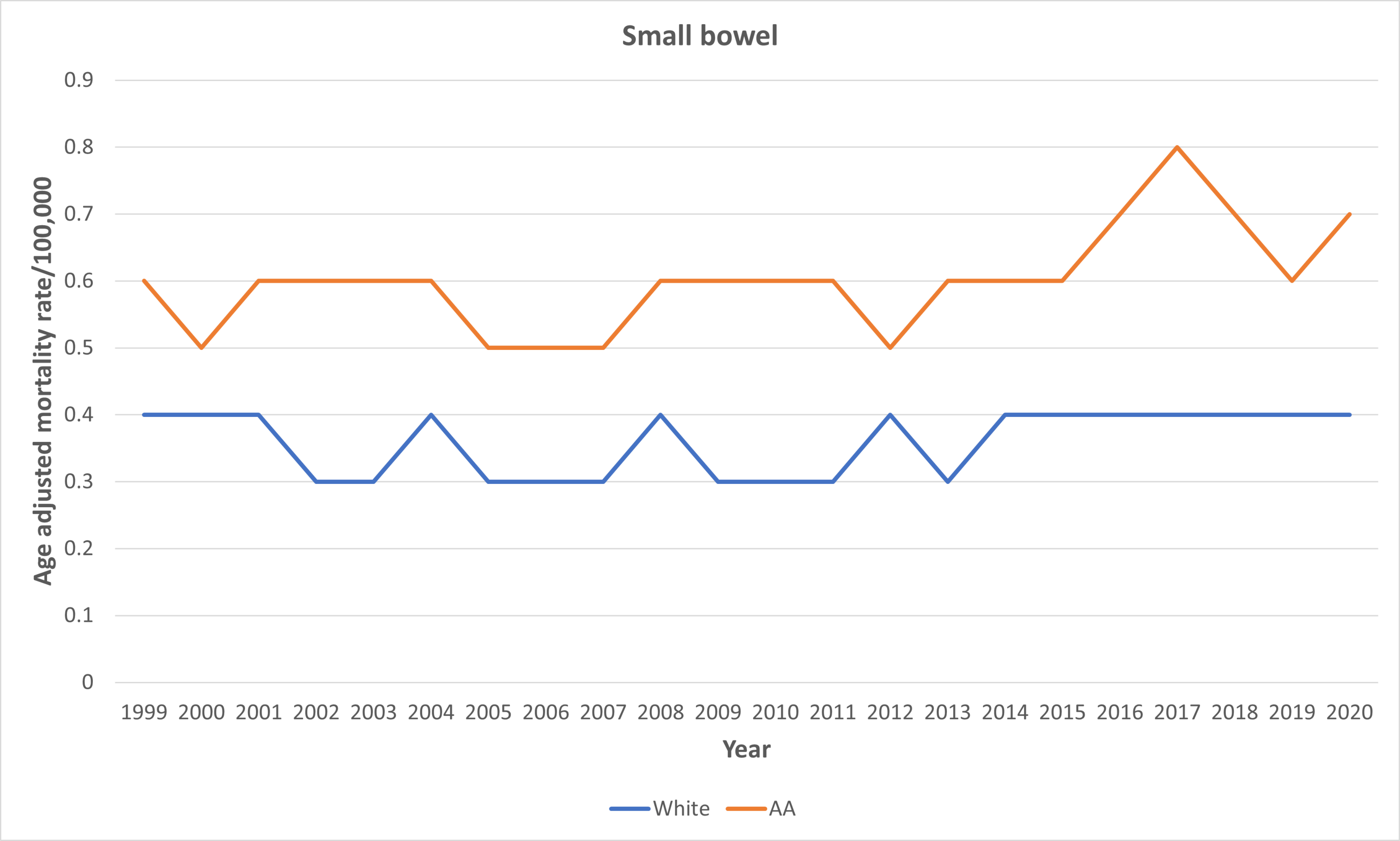
Small bowel
Age adjusted mortality rate/100,000
0.9
0.8
0.7
0.6
0.5
0.4
0.3
0.2
0.1
0
1999 2000 2001 2002 2003 2004 2005 2006 2007 2008 2009 2010 2011 2012 2013 2014 2015 2016 2017 2018 2019 2020
Year
White
AA

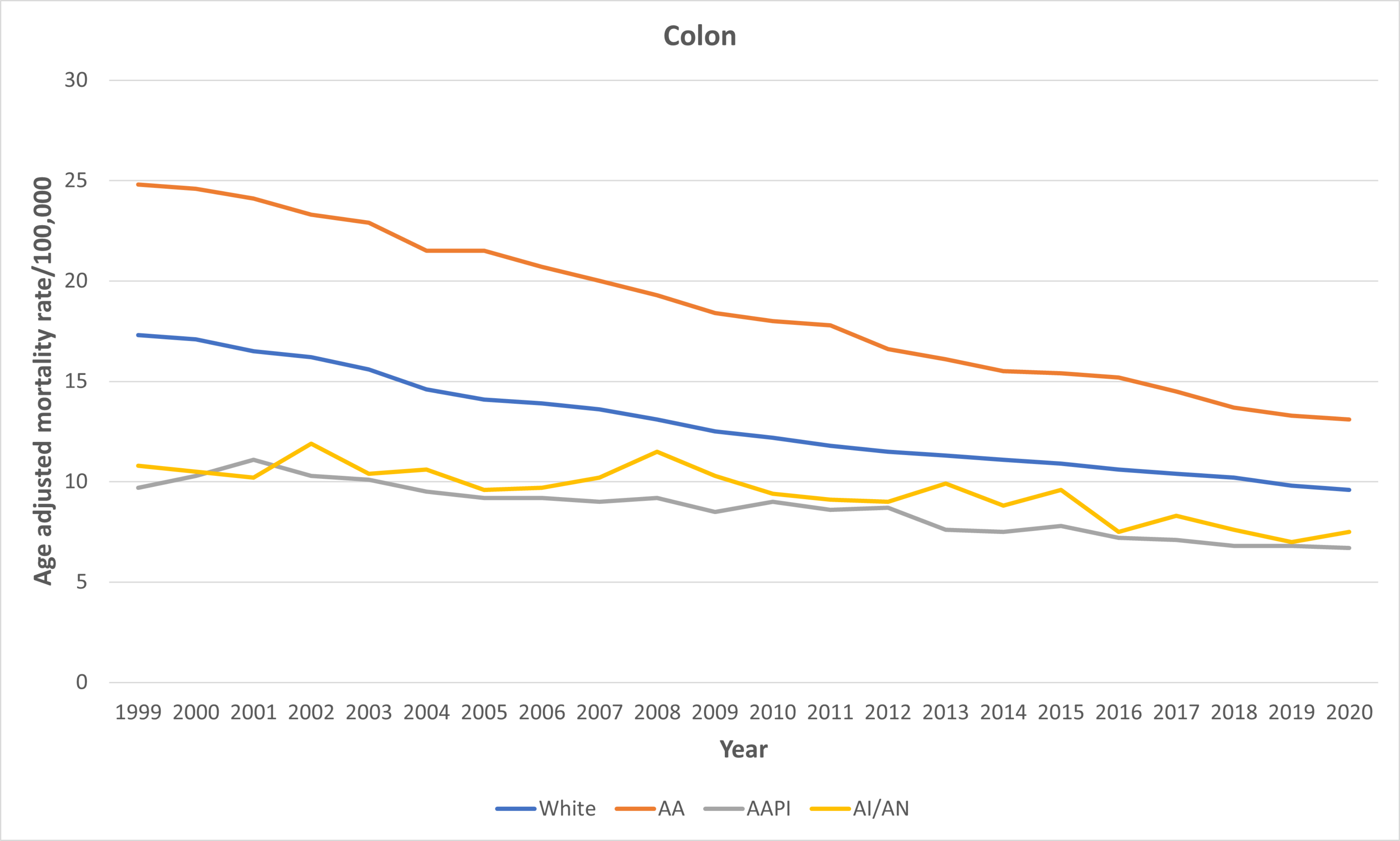

Colon
Age adjusted mortality rate/100,000
30
25
20
15
10
5
0
1999 2000 2001 2002 2003 2004 2005 2006 2007 2008 2009 2010 2011 2012 2013 2014 2015 2016 2017 2018 2019 2020
Year
White
AA
AAPI
AI/AN

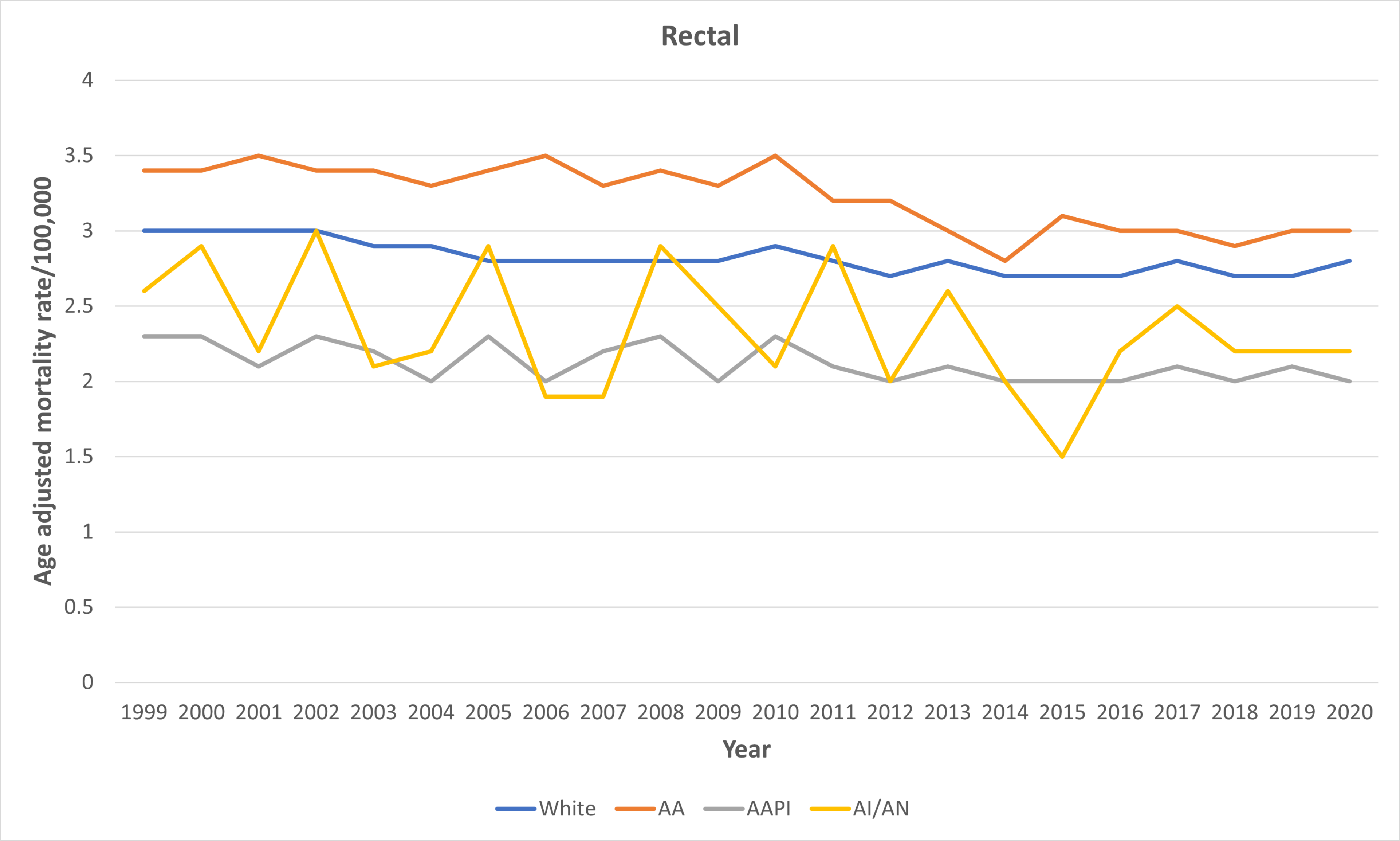

Rectal
Age adjusted mortality rate/100,000
4
3.5
3
2.5
2
1.5
1
0.5
0
1999
2000
2001
2002
2003
2004
2005
2006
2007
2008
2009
2010
2011
2012
2013
2014
2015
2016
2017
2018
2019
2020
Year
White
AA
AAPI
AI/AN

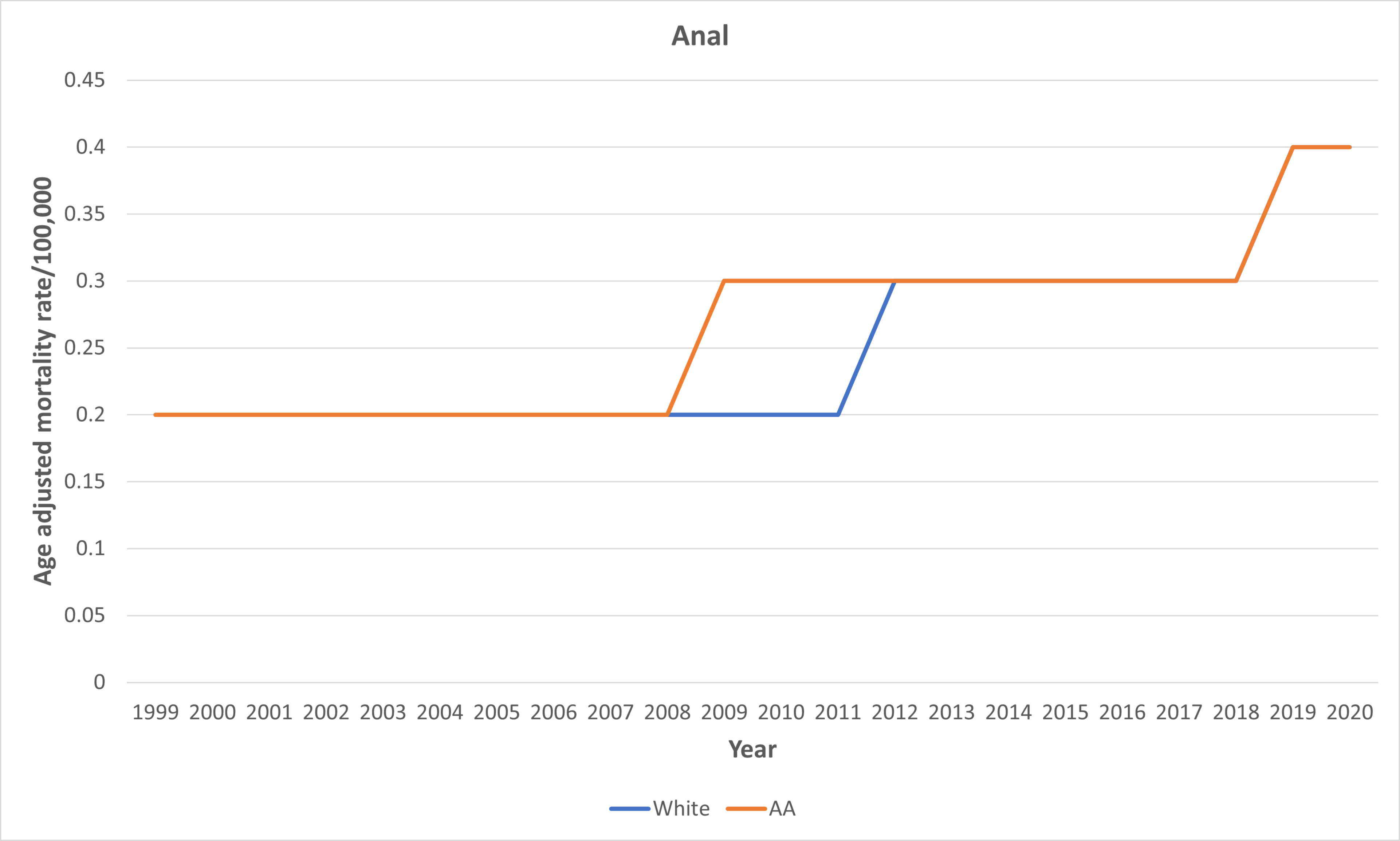
Anal
0.45
0.4
0.35
0.3
0.25
0.2
0.15
0.1
0.05
0
Age adjusted mortality rate/100,000
1999 2000 2001 2002 2003 2004 2005 2006 2007 2008 2009 2010 2011 2012 2013 2014 2015 2016 2017 2018 2019 2020
Year
White
AA

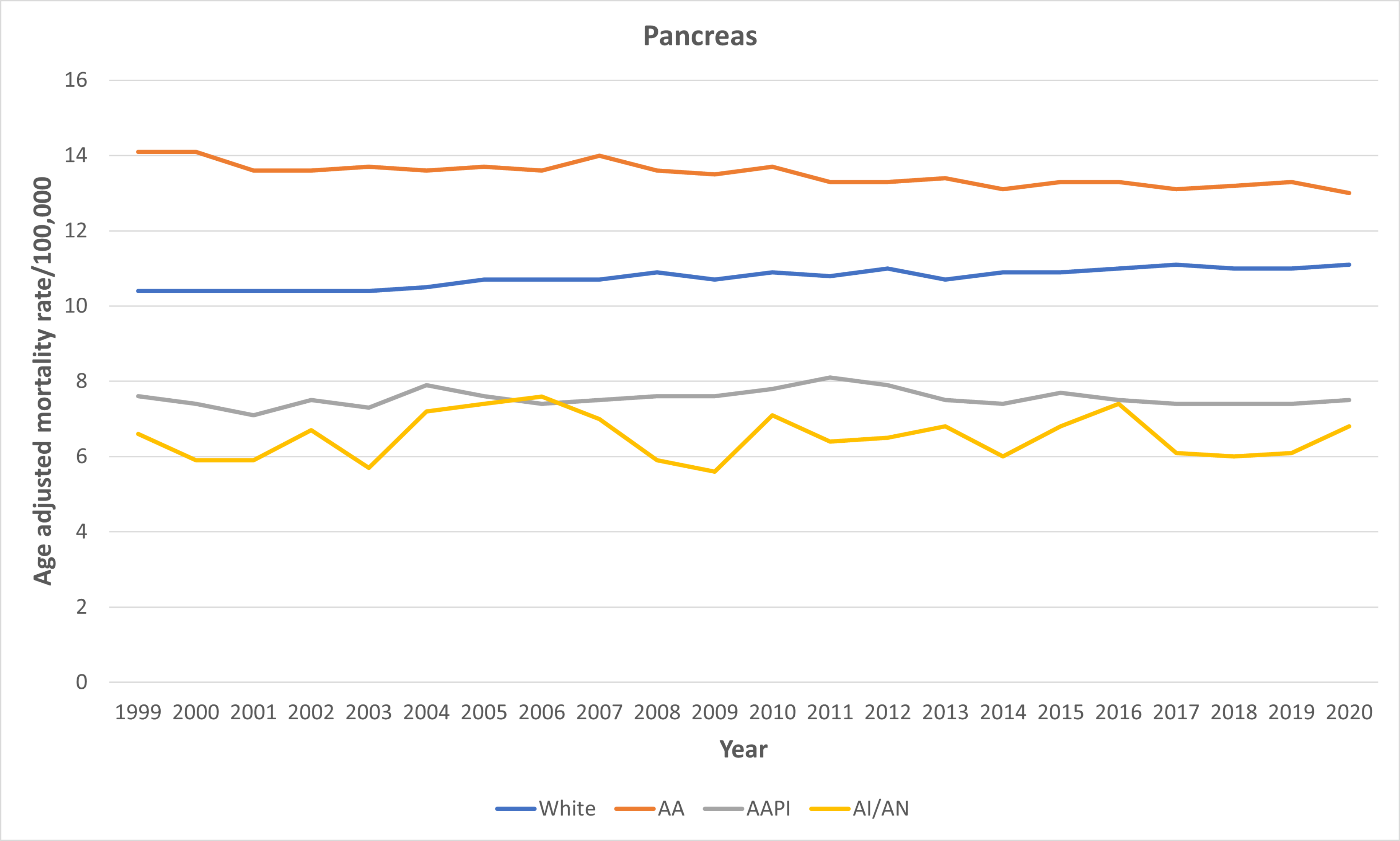

Pancreas
16
14
12
10
8
6
4
2
0
Age adjusted mortality rate/100,000
1999 2000 2001 2002 2003 2004 2005 2006 2007 2008 2009 2010 2011 2012 2013 2014 2015 2016 2017 2018 2019 2020
Year
White
AA
AAPI
AI/AN

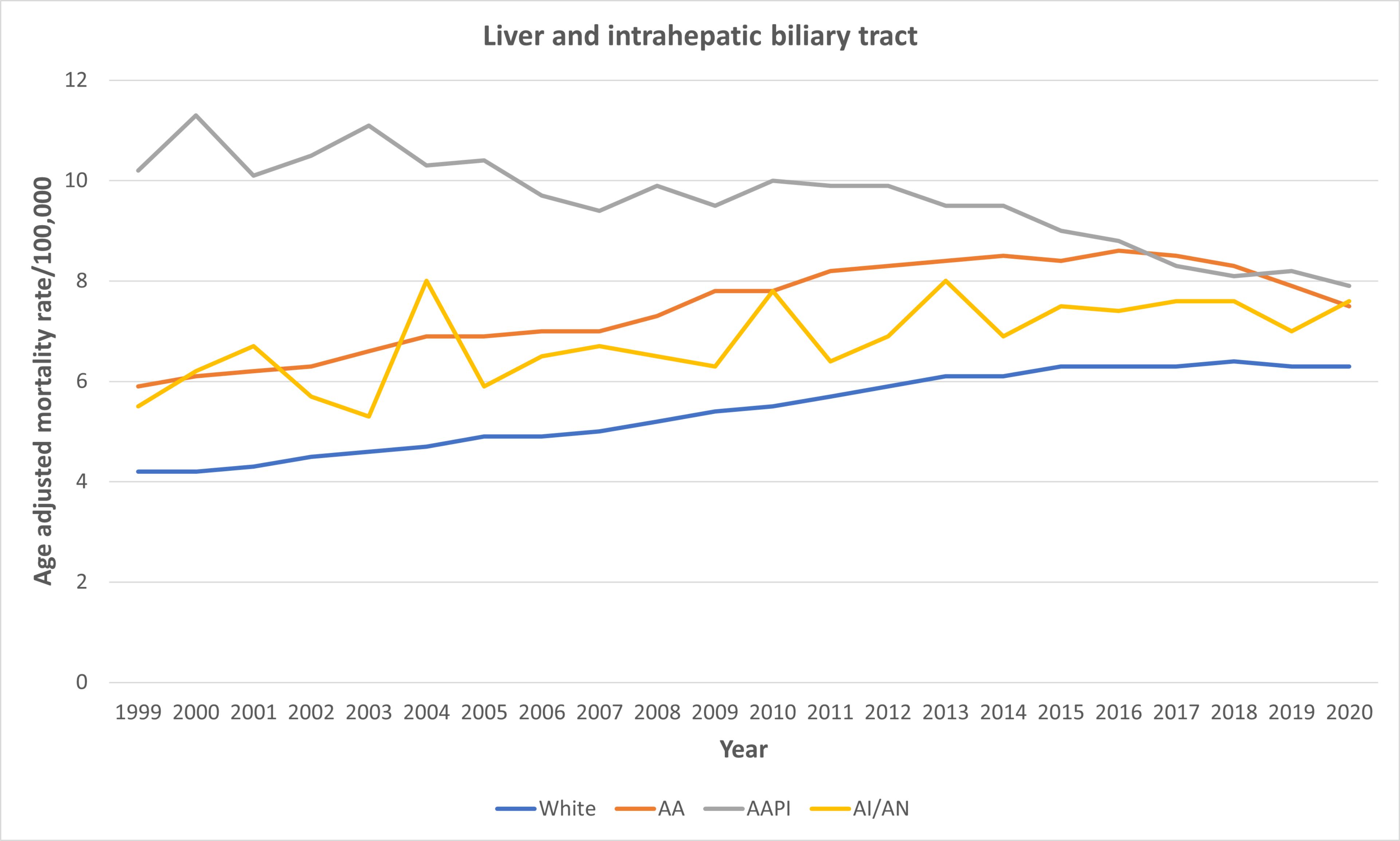
Liver and intrahepatic biliary tract
Age adjusted mortality rate/100,000
0
2
4
6
8
10
12
1999 2000 2001 2002 2003 2004 2005 2006 2007 2008 2009 2010 2011 2012 2013 2014 2015 2016 2017 2018 2019 2020
Year
White
AA
AAPI
AI/AN

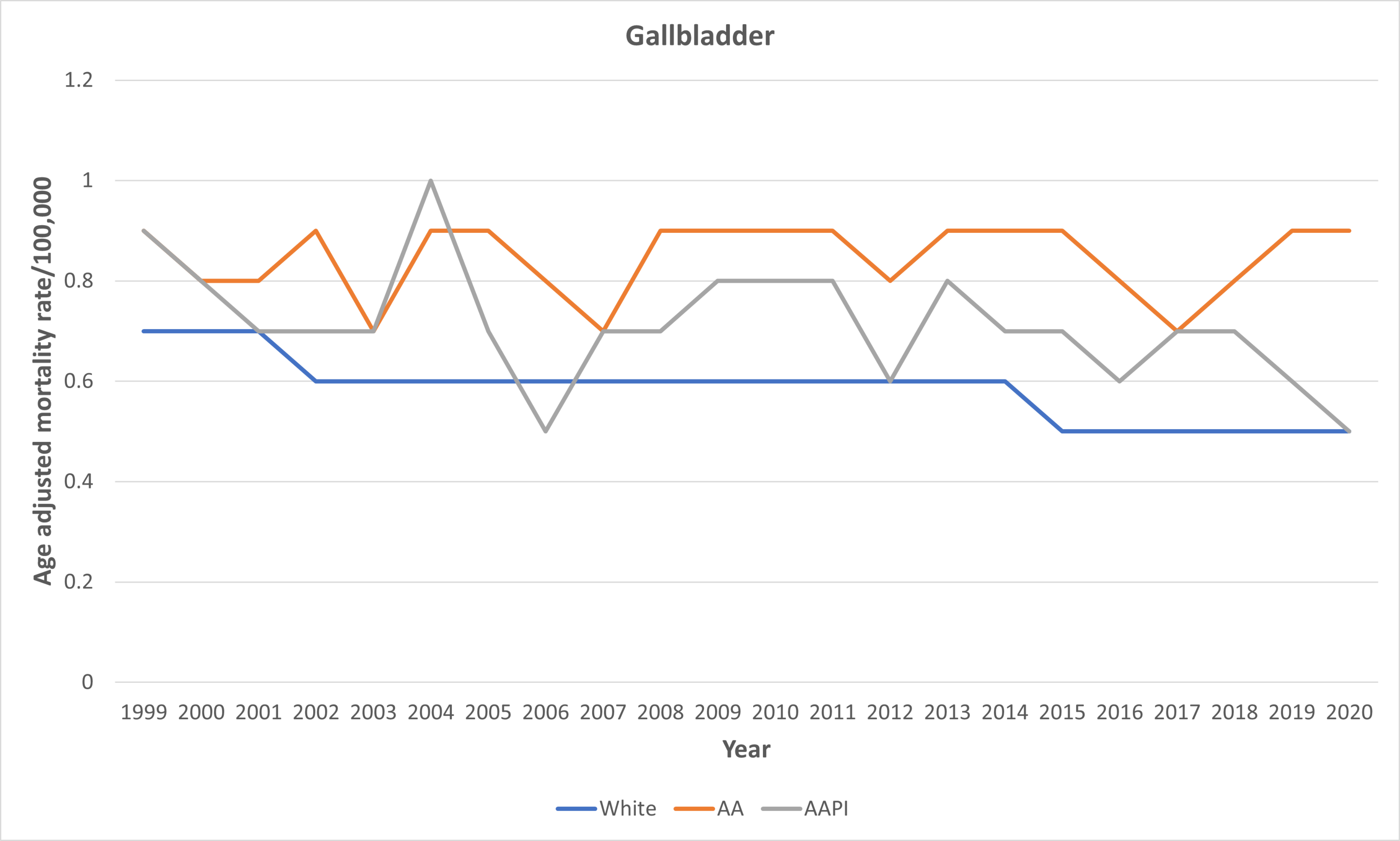
Gallbladder
1.2
1
0.8
0.6
0.4
0.2
0
Age adjusted mortality rate/100,000
1999 2000 2001 2002 2003 2004 2005 2006 2007 2008 2009 2010 2011 2012 2013 2014 2015 2016 2017 2018 2019 2020
Year
White
AA
AAPI

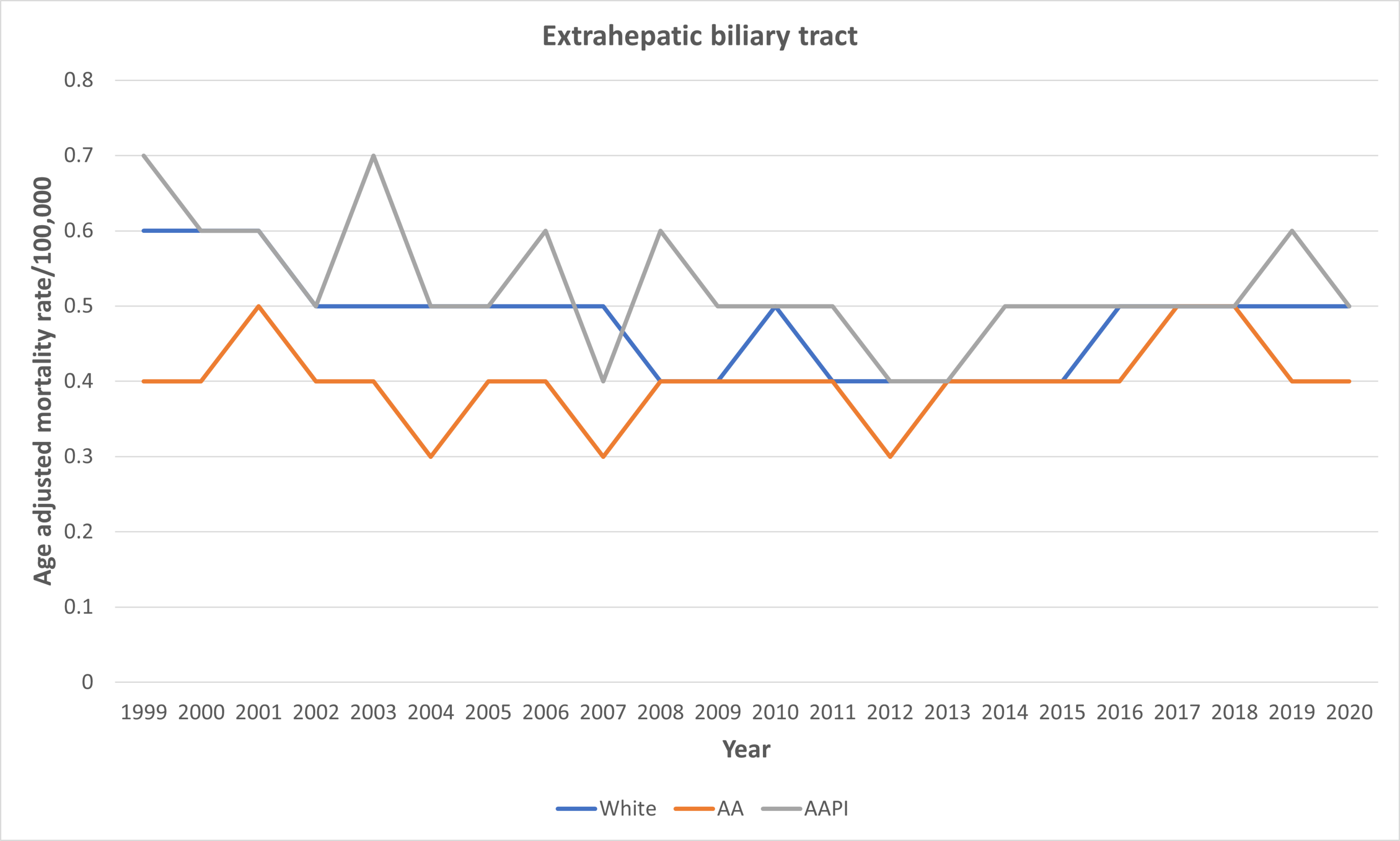
Extrahepatic biliary tract
Age adjusted mortality rate/100,000
0.8
0.7
0.6
0.5
0.4
0.3
0.2
0.1
0
1999 2000 2001 2002 2003 2004 2005 2006 2007 2008 2009 2010 2011 2012 2013 2014 2015 2016 2017 2018 2019 2020
Year
White
AA
AAPI
